# Supplementary material for: Gender differences in individual variation in academic grades fail to fit expected patterns for STEM
Source: Nat Commun. 2018 Sep 25;9:3777. doi: 10.1038/s41467-018-06292-0 (PMC6156605; doi:10.1038/s41467-018-06292-0)
Supplement: Supplementary file 1 — Supplementary Information [file 41467_2018_6292_MOESM1_ESM.pdf]

Supplementary Information (SI)

For:

Gender differences in individual variation in academic grades fail to fit expected patterns for STEM

Authors: **O'Dea, R.E. [1,2*], Lagisz, M. [1*], Jennions, M.D. [2], Nakagawa, S. [1]**

[1]Evolution & Ecology Research Centre, School of Biological and Environmental Sciences, University of New South Wales, Sydney, Australia
[2]Research School of Biology, Australian National University, Canberra, Australia
*These authors contributed equally to this work

Correspondence:
Correspondence can be addressed to:
Rose O'Dea
rose.eleanor.o.dea@gmail.com
Shinichi Nakagawa
s.nakagawa@unsw.edu.au

Supplementary Methods

Literature search

We collected data in two rounds. First, in March 2015, we collated the list of 309 studies and 502 effect sizes used in Voyer's systematic meta-analysis on gender differences in scholastic achievement[1]. We obtained copies of all but 14 studies from databases and libraries, and selected those with variance data for males and females (see below for eligibility criteria). Second, in June 2015, we searched for studies published since August 2011 (the time when Voyer's original search ended[1]) using three online literature databases. The exact search strings used were:

ERIC:
"school grade*" OR "school achievement*" OR "school mark*" OR "grade point average*"
Last 5 years, Peer reviewed only

SCOPUS:
TITLE-ABS-KEY ("school grade*" OR "school achievement*" OR "school mark*" OR "grade point average*") AND (LIMIT-TO(PUBYEAR, 2015) OR LIMIT-TO (PUBYEAR, 2014) OR LIMIT-TO (PUBYEAR, 2013) OR LIMIT-TO (PUBYEAR, 2012) OR LIMIT-TO(PUBYEAR, 2011))

ISI Web of Science:
TOPIC: (("school grade*" OR "school achievement*" OR "school mark*" OR "grade point average*")) Timespan: 2011-2015. Search language=Auto

Supplementary Fig. 3 shows the number of studies obtained by our search. Two investigators independently screened the titles and abstracts of these studies using *abstrackr* software[2], based on the detailed exclusion/inclusion criteria shown in Supplementary Fig. 3. Disagreements (17.7% of abstracts) were discussed and resolved. We then briefly screened the included studies for available data (presence of mean scores for males and females), before undergoing full-text screening and data extraction (described in the main text).

Mean-variance relationship

We observed strong relationships between mean grades and their variance estimates, for both genders, in the raw data (Supplementary Figs. 1 – 2), justifying the use of *lnCVR* as an effect size for statistically comparing grade variability between the sexes. However, when we restricted our plots to the data that could be considered most comparable due to using similar grading scales (0 – 5, or 0 – 100, North American schools), the relationship between means and SD disappeared (Supplementary Fig. 5).

Multiple imputations

We estimated missing values of students' mean age (37% missing) and study year (50% missing) using multiple imputation methods, implemented with the *mice* R package[3]. We used the following variables as predictors of missing values: publication year, school type, subject, minimum and maximum age of students in the cohort, and sex ratio of the sample cohort. We performed 100 imputation rounds (m = 100) for each data subset (full dataset, school subset, university subset, and data stacked by gender that was used for *lnCV* analyses). We ran meta-regression models that included study year, or mean student age, using the imputed datasets, and then pooled the results to average the estimates and compute the variances.

Data heterogeneity

We estimated total heterogeneity in the collected effect sizes as I^2 total values. I^2 statistics represent the proportion of the total variation that is due to the between-study variation (i.e. the true heterogeneity) rather than due to sampling errors associated with each effect size. Total heterogeneity values in the whole dataset and

both school and university data subsets were very high ($I^2_{\text{Total}} > 98\%$; Supplementary Table 19), warranting looking for predictor variables (moderators) to explain variation among effect sizes.

University data subset and full data set

Meta-analytical and meta-regression models reported for the school data subset in the main text were also run on the university subset and the full data set (school and university data pooled). We found no effect of study year (Supplementary Tables 5-6). The gender difference in mean grades decreased significantly as students grew older, but this difference was only significant in the full data set (Supplementary Table 6). The gender difference in grade variability did not change with student age (Supplementary Table 6), but total variability decreased with age among both girls and boys at university, and only among boys in the full data set (Supplementary Table 7). When study subject was considered, the gender gap in mean grades was observed in the university subset and the full data set for every subject except "Other/NR" (Supplementary Table 8). Lower female variability was significant in every subject for the full data set, but only significant in STEM and Global subjects in the university subset (Supplementary Table 9). Additionally, data from the university was predominantly for "Global" grades; there were only 13 data points for science, 10 for maths and 4 for language. These low sample sizes resulted in broad confidence intervals for moderator analyses on the effect of subject (e.g., Supplementary Fig. 10E), and sensitivity to outliers. In particular, one influential study for language grade variability in the university subset strongly influenced the difference in variability between STEM and non-STEM subjects, causing there to be no significant differences in variability in non-STEM (Supplementary Table 9).

Robustness of results

1. We considered an alternative way to classify school/study subjects, following the approach in[1], with six subject categories: global, science, math, language, social sciences and other. We re-ran appropriate meta-regression models using this moderator. The results are qualitatively similar to these obtained with our original subject classification (STEM, non-STEM, Global, Other) and are presented in Supplementary Fig. 10 and Supplementary Tables 16 – 19.

2. We tested the robustness of our meta-analytical and meta-regression results using the *robumeta R* package[4] for robust variance estimation (RVE). The results of these analyses are very similar to those obtained using the *robust* function in the *metafor R* package, and they are available in this online repository: https://osf.io/ejqm4/.

3. We constructed multivariate (full) meta-regression models to simultaneously test the effects of three moderators: study year, mean student age, and subject type (classified as in Voyer & Voyer 2014). The outcomes from these models (Supplementary Tables 21 – 23) match the patterns observed in the univariate models (no effect of study year or age, higher female mean grades, and lower female grade variability, especially for Global and Language grades in the school subset).

4. We also used Bayesian meta-regression models, with all three moderators (study year, mean student age, and subject type) included. These models were run using *MCMCglmm* package[5] with default (non-informative) prior, except for university subset where a inverse-Gamma prior was used for the variance component for comparison ID, and parameter expanded prior for the variance component for cohort ID. For each model we ran 3 chains, with 110,000 iterations, 10,000 burn-in periods, and thinning by every 1000. The results obtained were qualitatively similar to the

results from the maximum-likelihood (*metafor*) univariate and multivariate (full) models (Supplementary Tables 24 – 25).

Publication bias analyses

Publication bias can be present in a meta-analytic data set when experimental results that show strong effects are preferentially published. To test for publication bias in our data set we first visually inspected funnel plots for asymmetry in the distribution of the collected effect sizes, and then performed Egger's regression on residuals of effect-sizes from meta-analytic models and sampling errors[6,7]. Residuals were calculated from full Bayesian models including study year, student mean age and subject as moderators. Visual inspection of funnel plots (Supplementary Figs. 6 - 7) and results of Egger regression do not indicate publication bias in the school subset, but there is evidence of publication bias in the university subset (Supplementary Table 25). We found no effect of publication year on our *lnRR* and *lnCVR* values in univariate models (Supplementary Table 12).

Inferring grade distributions overlaps

We used simulations to translate the estimated effect sizes for the ratios between mean grades and grade variabilities between the sexes into predicted distributions of student's academic abilities. We assumed that school grades represent student's ability for future success in the academic discipline[8], and we assumed normal distributions of grades within populations. From the North American school data with grading scale 0-5 we calculated mean and SD of boys' grades for STEM and non-STEM subjects. We then obtained respective mean and SD of girls' grades by utilising mean effect size estimates, expressed as percentages. Using means and SD

values of boys' and girls' grades we generated a sample of 1,000,000 boys and 1,000,000 girls. The sample was used to plot distributions of individual grades (Fig. 3A and 3B), and to estimate the proportion of girls to boys in the top portion of the distribution, for divisions of 0.1% (i.e. 1000 divisions, from 0.001 to 1) (Fig. 3C).

Comparison of STEM and non-STEM grades within the same cohort

In our school data subset, we found girls had a smaller mean advantage in STEM compared to non-STEM subjects. Does this imply that girls are receiving lower grades in STEM than non-STEM, or are boys receiving higher grades in STEM than non-STEM? We cannot test this question directly, because our data does not contain individual-level data, but we can test it at the level of a cohort. To test whether, on averages, students in a class or school are receiving higher grades in STEM compared to non-STEM, we reduced our data to studies that presented mean school grades in STEM and non-STEM subjects for boys and girls from the same cohort (30 cohorts from 25 studies). We then computed the log response ratio (*lnRR*) as the logged ratio between mean STEM and mean non-STEM grades.

We performed meta-analysis on the within-cohort difference between mean STEM and non-STEM grades, and a meta-regression with sex as a moderator. The results of these analyses are shown in Supplementary Table 11. Overall, there was a non-significant trend for STEM grades to be 3.9% lower than non-STEM grades. This trend was entirely driven by girls, whose STEM grades were significantly 6.7% lower than their non-STEM grades. Boys' grades showed no significant difference between STEM and non-STEM.

Direct comparison to Voyer & Voyer 2014: Standardised Mean Difference

In addition to analysing gender differences in mean grades using *lnRR*, we also ran analyses using the standardised mean difference (*SMD*). This allows more direct comparison with the results of Voyer's[1] original meta-analysis, which used Cohen's *d*[9] as the effect size. We have used a modified version of Cohen's *d*, called Hedge's *d* or *g*[10], which has a bias correction for small sample sizes. We calculated *SMD* and its sampling variance, s^2_{SMD}, as:

$SMD = \frac{\bar{x}_f - \bar{x}_m}{s_{\text{pooled}}} J$ (Supplementary Equation 1)

$J = 1 - \frac{3}{4(n_f + n_m - 2) - 1}$ (Supplementary Equation 2)

$s_{\text{pooled}} = \sqrt{\frac{(n_f - 1)s_f^2 + (n_m - 1)s_m^2}{n_f + n_m - 2}}$ (Supplementary Equation 3)

$s^2_{SMD} = \frac{n_C + n_E}{n_C n_E} + \frac{SMD^2}{2(n_C + n_E)}$ (Supplementary Equation 4)

Where:

$\bar{x}_f$ and $\bar{x}_m$ = the mean grade of female and male students, respectively

s_m^2 and s_f^2 = the variance in grades of female and male students, respectively

n_m and n_f = the number of male and female students in each sample, respectively

Our results are similar to Voyer's[1] in both magnitude and significance, with the exception that course material classified as "Other/NR" showed no significant mean difference (our results: Supplementary Table 16; Voyer's results: Table 2, p.1189[1]).

Supplementary Note 1

Do gender differences vary with racial composition?

Following Voyer's[1] methods, for the samples from the United States we coded the racial composition of each cohort if the original study reported a >75% racial majority for the student population (for coding details, see Supplementary Table 1). Twelve studies reported a majority Black/African American racial composition (n = 15 effect sizes), and eighteen studies reported a White/Caucasian majority (n = 36 effect sizes). No studies reported an Asian American majority, and only two studies (n = 2 effect sizes) reported a Latino/Hispanic majority, providing insufficient data for a comparison. Therefore, to test for the effects of racial composition on gender differences in school grades, we used the racial composition category of White/Black as a moderator variable in univariate meta-regression models. We found no significant differences in gender differences in either mean or variance (Supplementary Table 13).

Supplementary Note 2

Analysis of test scores – 2015 PISA

To explore whether gender differences in variability across STEM and non-STEM are broadly applicable to school achievement, and not confined to school grades which tend to favour girls, we analysed data from the 2015 Programme for International Student Assessment (referred to as PISA hereafter). PISA is an international measurement of achievement on standardised tests by 15-year-old students.

We downloaded results tables for test performance in reading, mathematics, and science subjects, from the PISA 2015 Results (Volume I)[11], and extracted the means

and standard deviation in achievement for boys and girls in each jurisdiction (country). We obtained the corresponding sample sizes from separate tables, available within the "Questionnaire items" from the Compendia on the PISA 2015 Database webpage[12]. To calculate sample sizes we used the number of "valid" students for each subject type, and the given percentage of boys and girls within each jurisdiction.

Overall, the PISA dataset summarised test scores in maths, reading and science for 226,131 female and 226,480 male students. The students were tested from 64 jurisdictions. The minimum and maximum number of students in a jurisdiction was 3,371 and 23,141, respectively.

To test for gender differences in means we used the same metric as our main meta-analysis (*lnRR*). To test for differences in variance we used a different metric, because there was no consistent mean-variance relationship in the PISA dataset (in contrast to our main dataset). We therefore used the log variability ratio (*lnVR*) and its sampling variance ($s^2_{\ln VR}$) to test for gender differences in variance[13], where:

$lnVR = \ln\left(\frac{s_f}{s_m}\right) + \frac{1}{2(n_f-1)} - \frac{1}{2(n_m-1)}$ (Supplementary Equation 5)

$s^2_{lnVR} = \frac{1}{2(n_f-1)} + \frac{1}{2(n_m-1)}$ (Supplementary Equation 6)

We also directly modelled between-subject differences in variability by testing for the moderating effects of sex and subject on the logged standard deviation (*lnSD*) in test scores[14]:

$lnSD = \ln s + \frac{1}{2(n-1)}$ (Supplementary Equation 7)

$s^2_{lnSD} = \frac{1}{2(n-1)}$ (Supplementary Equation 8)

We fitted meta-analytic models to each effect size with the same approach as our main analysis (using the *rma.mv* and *robust* functions from the *metafor* package (v. 2.0.0) in *R* (v. 3.4.3)[15]). We accounted for non-independence arising from multiple effect sizes from the same study by fitting the ID of the jurisdiction and a comparison ID as random effects. We modelled sampling variances with a covariance matrix, assuming a 0.5 correlation between variances arising from the same jurisdiction. To test for differences between STEM and non-STEM subjects, we fitted univariate meta-regression models by including subject as a fixed effect, where subject was either STEM (maths and science) or non-STEM (reading).

PISA Results

Full results are shown in Supplementary Table 14. Overall, girls sitting the 2015 PISA received 2% higher scores than boys ($lnRR_{\text{overall(mean)}}$ CI: 1.4% to 2.7%; Supplementary Fig. 11A), with 6.4% less variation among girls than among boys ($lnVR_{\text{overall(variance)}}$: CI: 5.6% to 7.2%; Supplementary Fig. 11B).

Girls' small advantage in PISA tests scores was entirely driven by a 6.9% advantage in non-STEM ($lnRR_{\text{STEM(mean)}}$ CI: 6% to 7.7%. In contrast, girls' showed a non-significant 0.3% *disadvantage* in STEM ($lnRR_{\text{non-STEM(mean)}}$ CI: -0.9% to 0.2%; Supplementary Fig. 11A).

While girls' test scores were significantly more consistent across subjects, this variability gap was no different between non-STEM and STEM subjects ($lnVR_{\text{non\_STEM STEM diff}}$ CI: -0.7% to 0.3%). When looking at the variability for girls and boys separately, both girls' and boys' scores were more variable in non-STEM than STEM subjects, but the difference was smaller for girls (Supplementary Fig. 11C, Supplementary Table 14).

Together these results suggest that the shape of the achievement distributions was consistent, but in STEM subjects boys' test scores shifted to the right.

Supplementary Figures

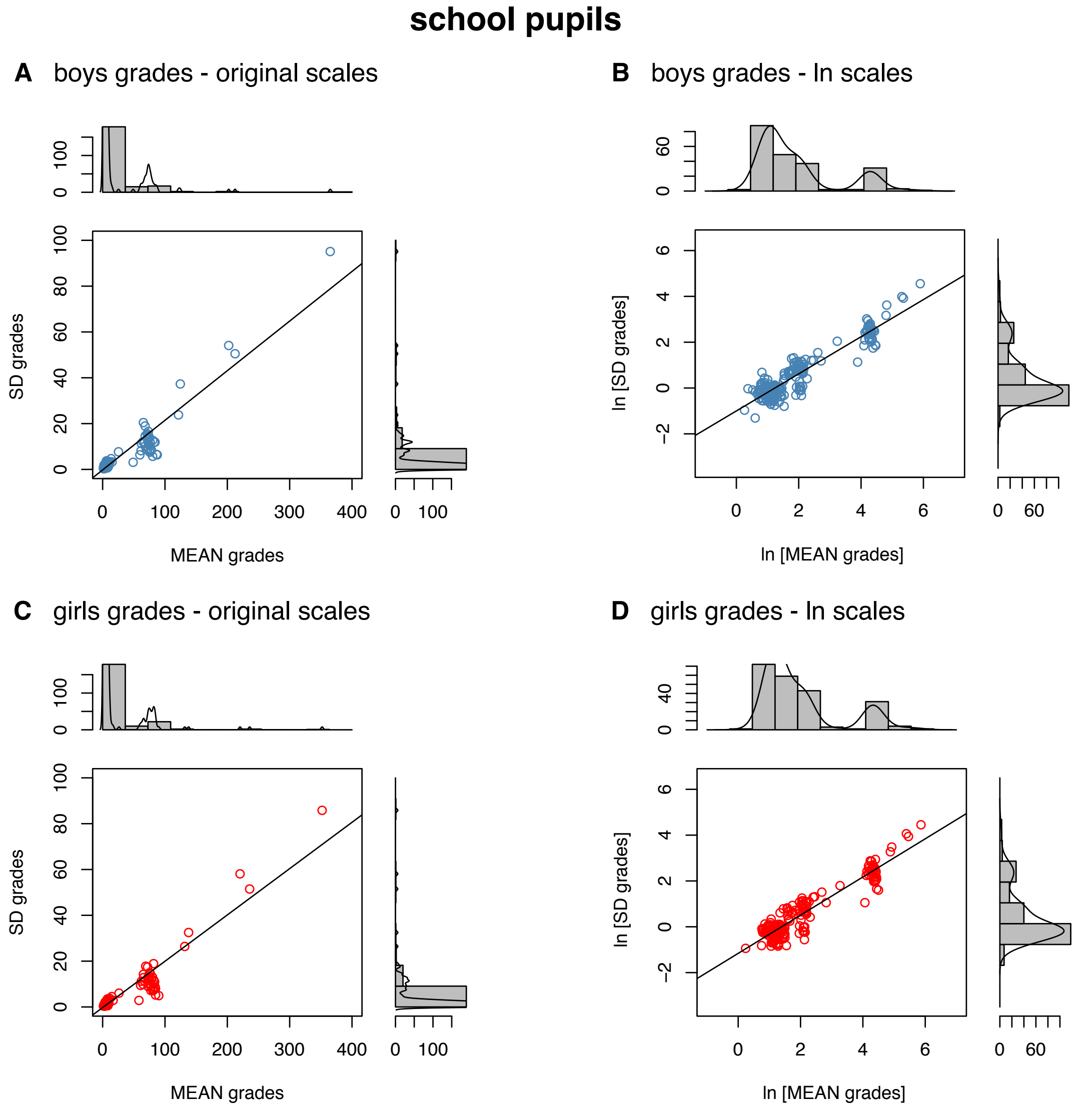

Supplementary Figure 1

School data subset – relationships between the means and their standard deviations (SD) of grades of boys (**A**, **B** - blue) and girls (**C**, **D** - red), on original and log scales. Distributions of means and standard deviations, presented as side histograms, indicate that a few different grading scales were used for grading students in the original studies.

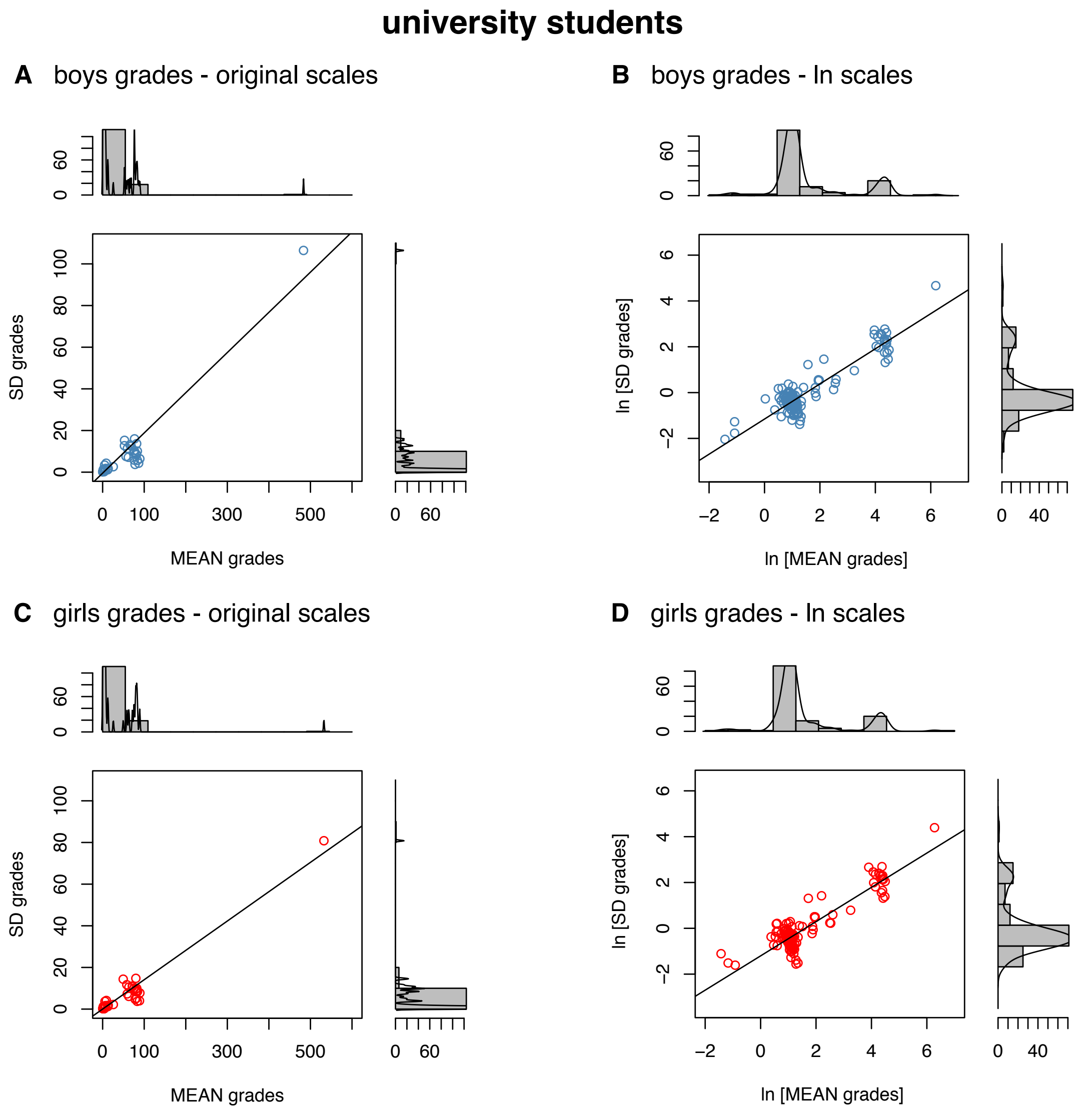

Supplementary Figure 2

University data subset – relationships between the means and their standard deviations (SD) of grades of boys (**A**, **B** - blue) and girls (**C**, **D** - red), on original and log scales. Distributions of means and standard deviations, presented as side histograms, indicate that a few different grading scales were used for grading students in the original studies.

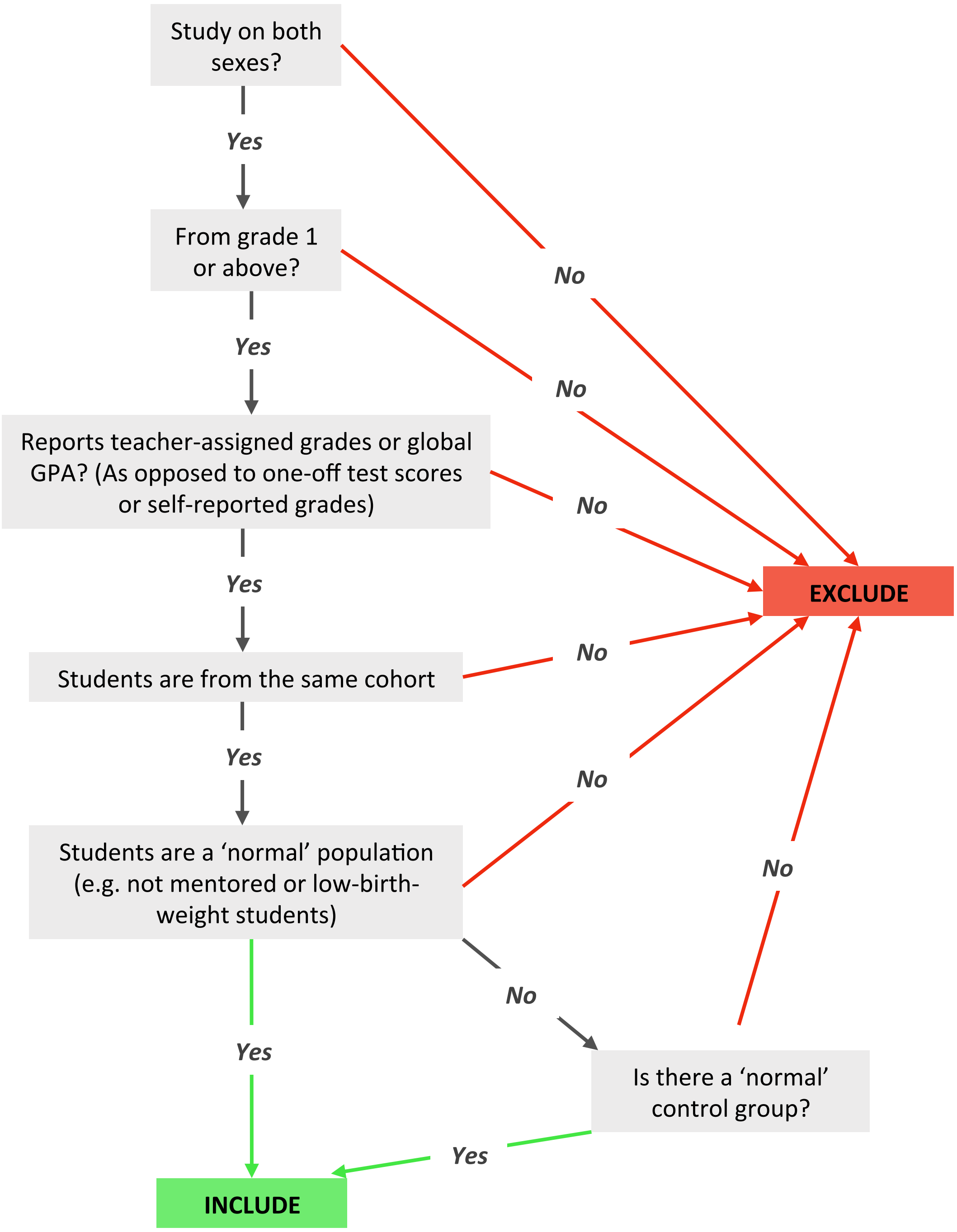

Supplementary Figure 3

Inclusion/exclusion decision tree with criteria used to evaluate found studies for inclusion in the meta-analysis.

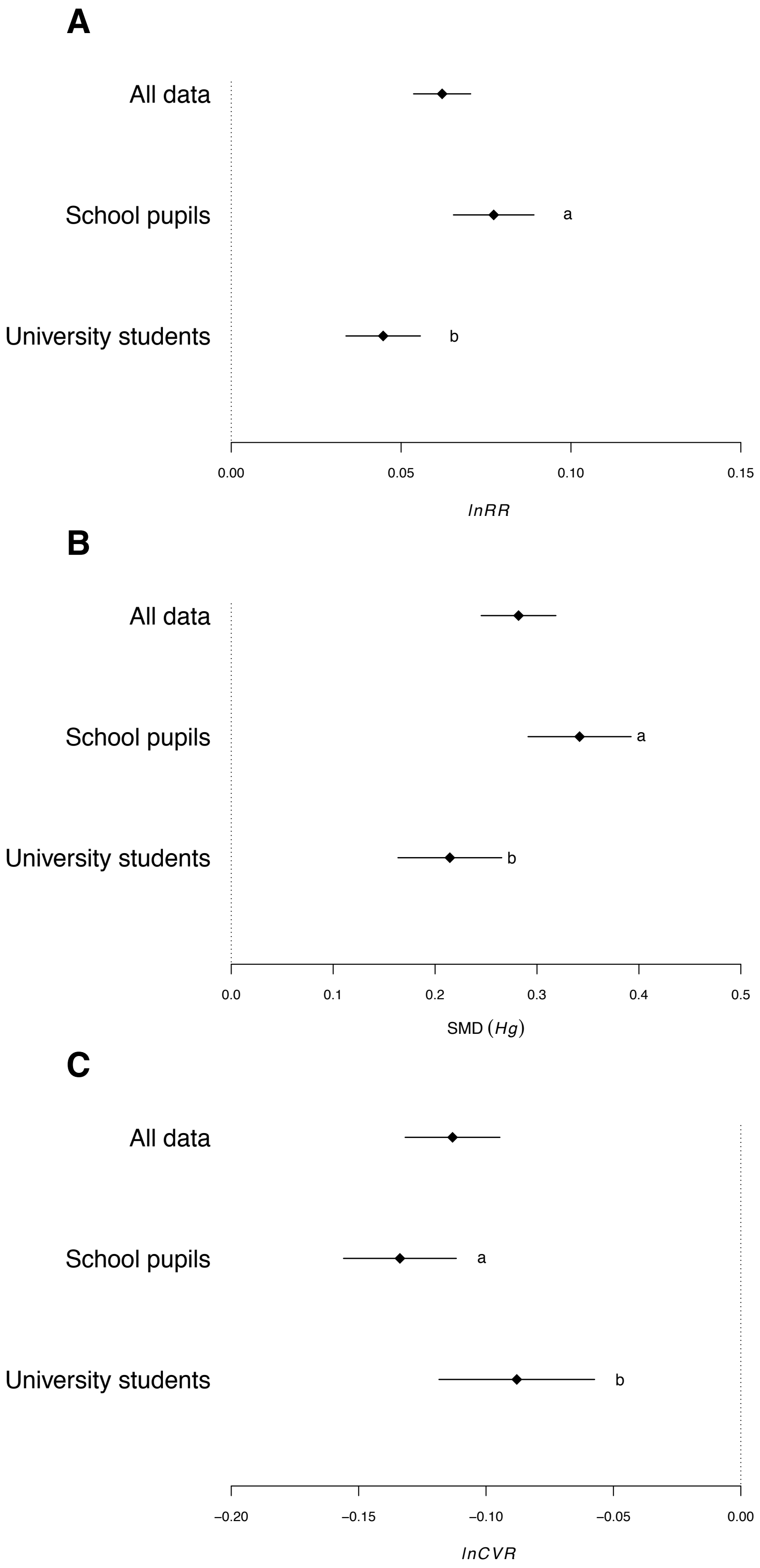

Supplementary Figure 4

Meta-analytic estimates of mean effect sizes, and 95% confidence intervals, for gender differences in grades in the whole sample (All data), and in the school and

university data subsets. (**A**) and (**B**) Positive *lnRR* and *SMD* estimates indicate lower boys' mean grades in comparison to girls' mean grades. (**C**) Negative *lnCVR* estimates indicate greater grade variation for boys in comparison to girls, with the gender gap being larger at school than at university. Lowercase 'a' and 'b' letters indicate that school and university mean estimates are statistically different, with a larger gender gap at school than at university.

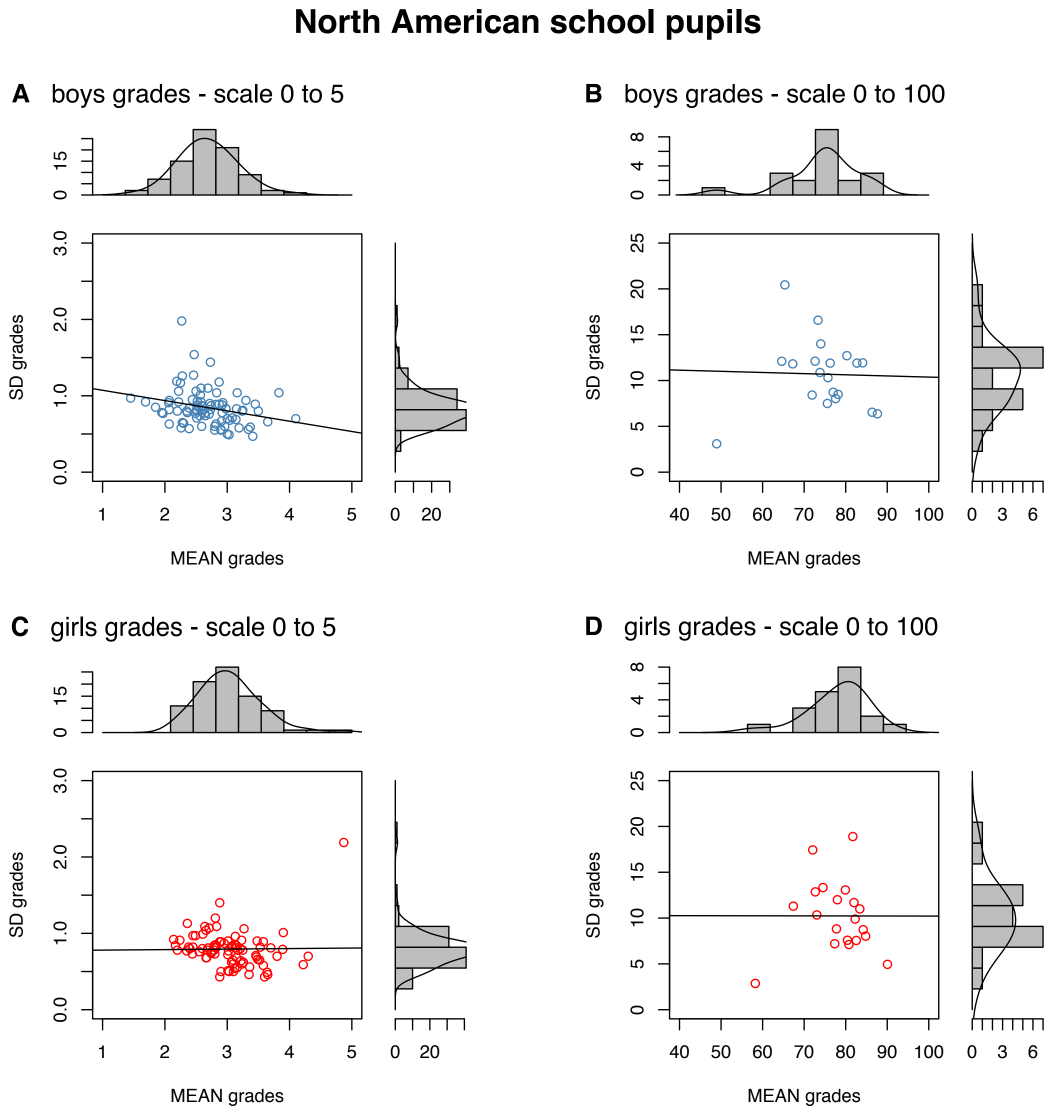

Supplementary Figure 5

School data subsets for North American students graded on scales 0-5 (82 effect sizes) and 0-100 (23 effect sizes) – relationships between the means and their standard deviations (*SD*) of grades of boys (**A**, **B** - blue) and girls (**C**, **D** - red), on both scales. Distributions of means and standard deviations presented as side histograms do not indicate ceiling effect in these data subsets.

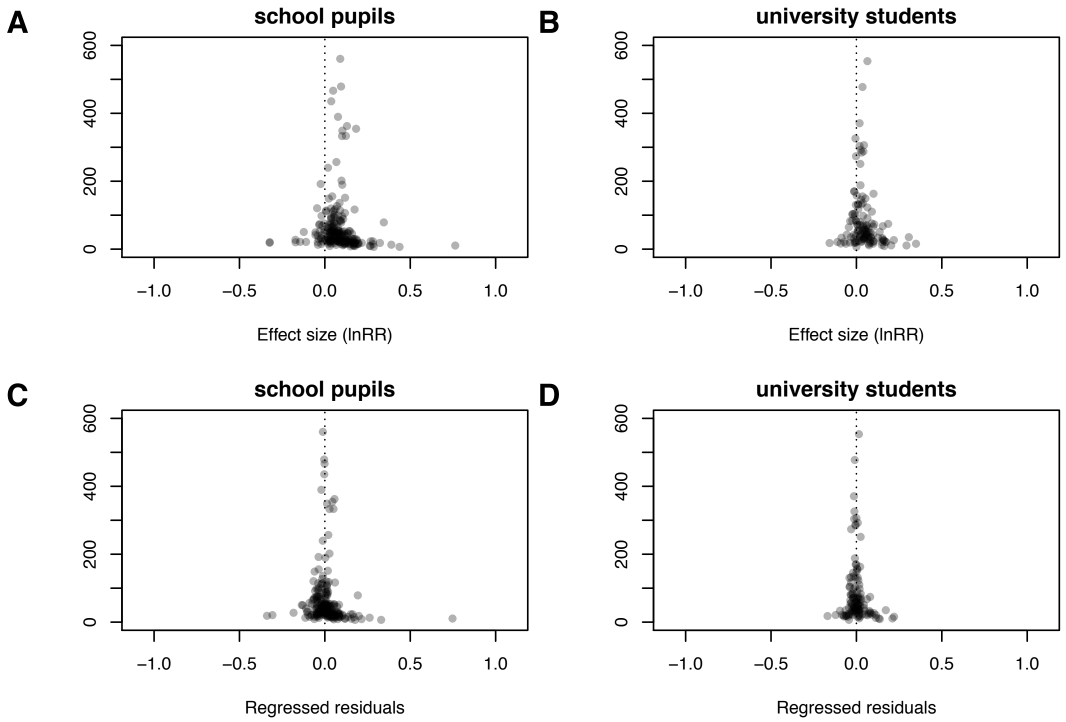

Supplementary Figure 6

Funnel plots for differences in mean grades of boys and girls (*lnRR*). Panels **A** and **B** are based on raw effect sizes and their precision (inversed square root of standard errors). Panels **C** and **D** are based on the residual values from the full meta-regression models, containing all moderators, and their precision.

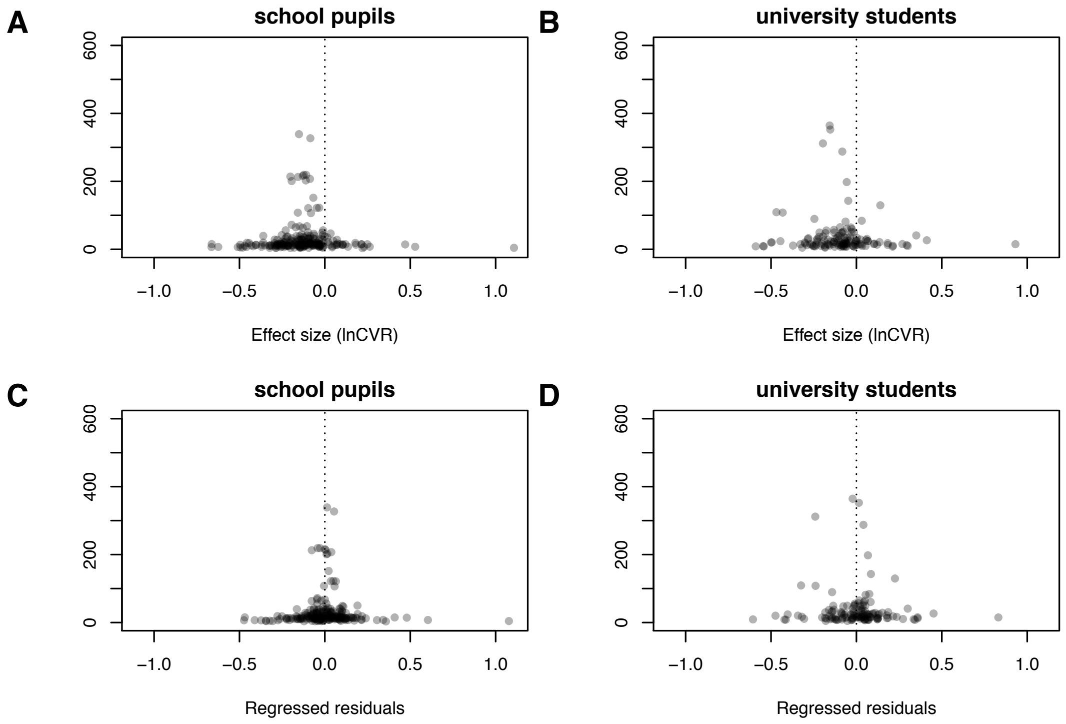

Supplementary Figure 7

Funnel plots for differences in variation of grades of boys and girls (*lnCVR*). Panels **A** and **B** are based on raw effect sizes and their precision (inversed square root of standard errors). Panels **C** and **D** are based on the residual values from the full meta-regression models, containing all moderators, and their precision.

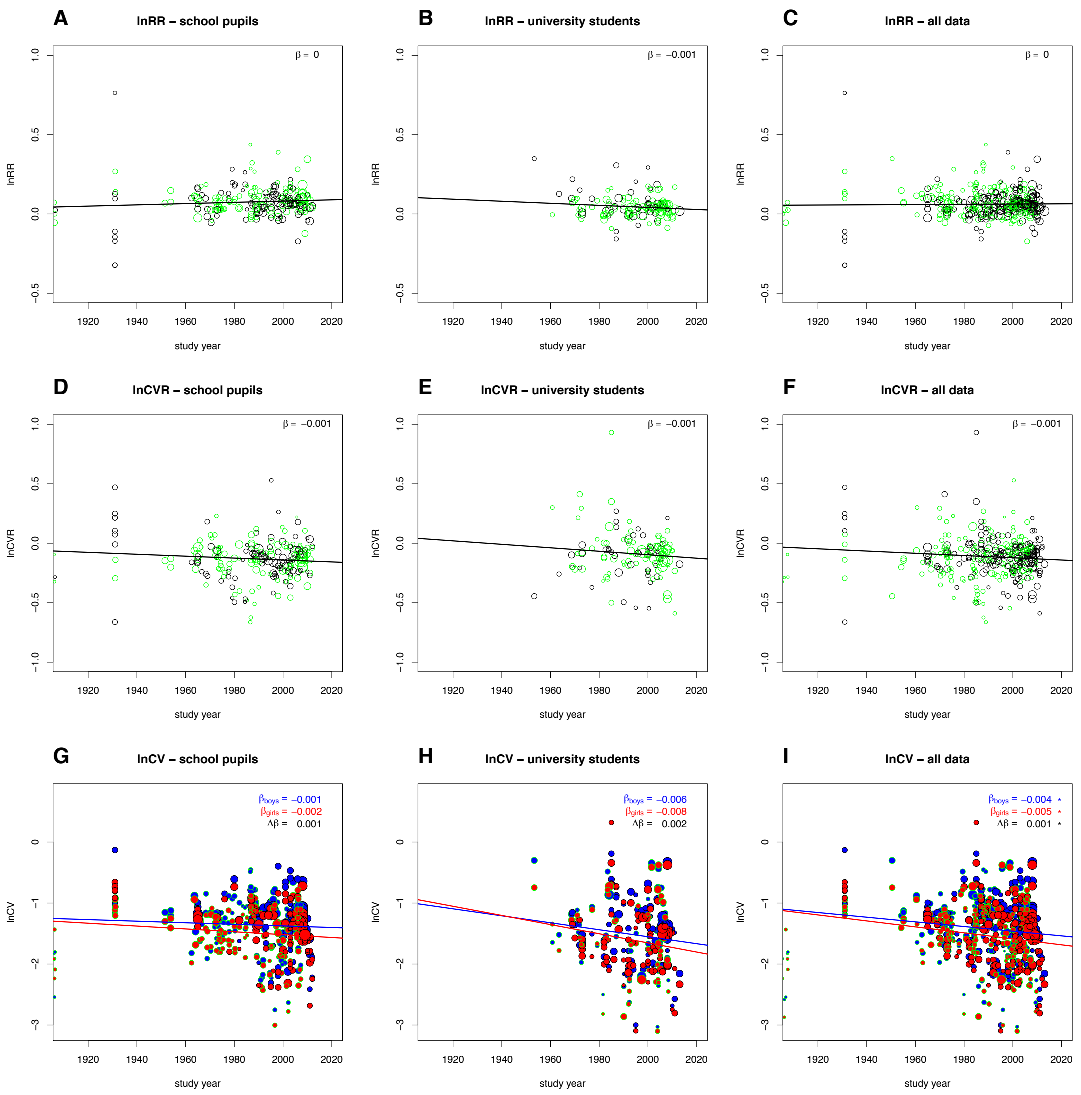

Supplementary Figure 8

Scatter plots for effect sizes against study year for the school subset (panels **A**, **D** and **G**), the university subset (**B**, **E** and **H**), and the whole dataset (**C**, **F** and **I**). Green circles represent imputed study year data. In panels **G**, **H** and **I**, blue points represent data for boys' grades, and red points represent data for girls' grades.

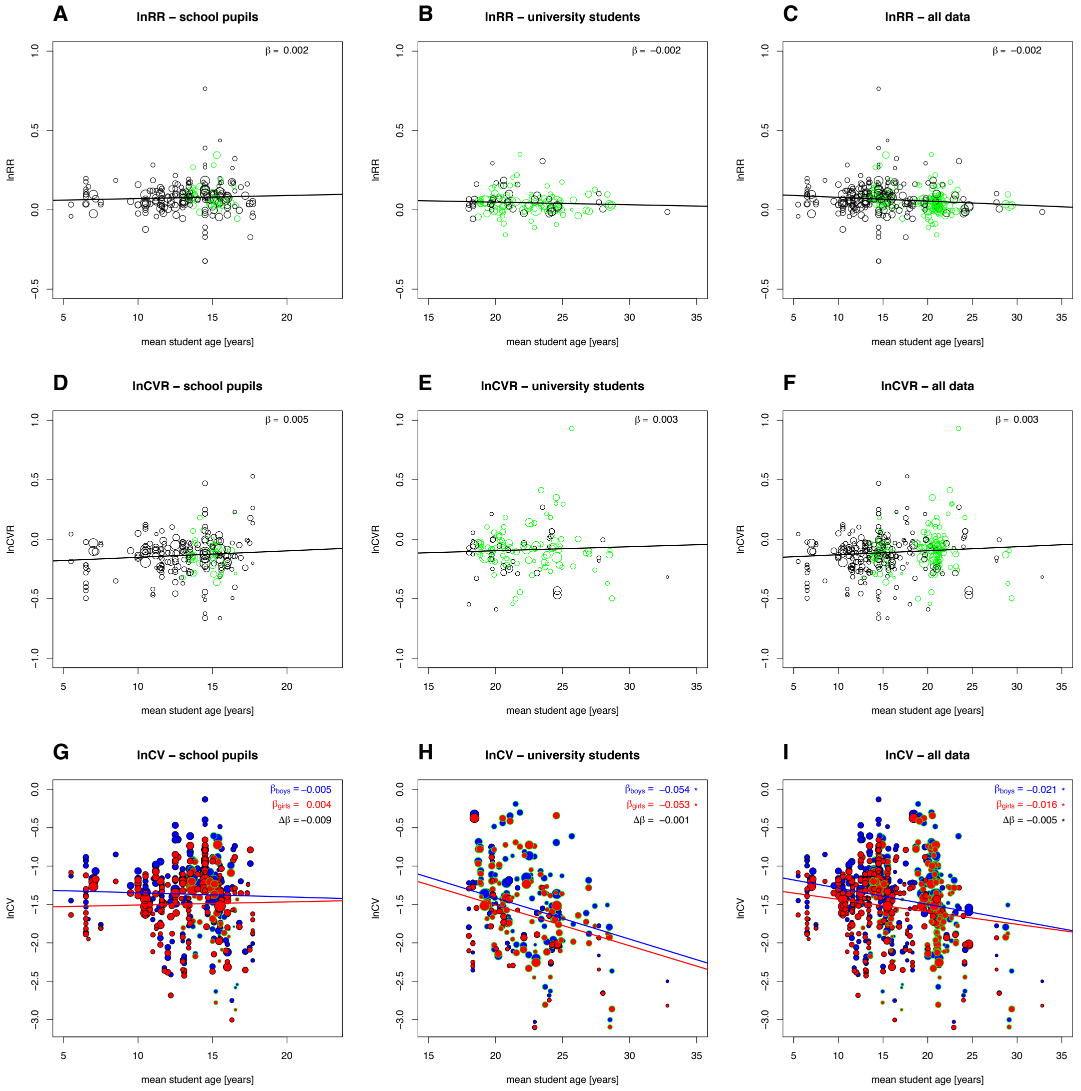

Supplementary Figure 9

Scatter plots for effect sizes against mean student age for the school subset (panels **A**, **D** and **G**), the university subset (**B**, **E** and **H**), and the whole dataset (**C**, **F** and **I**). Green circles represent imputed mean age data. In panels **G**, **H** and **I**, blue points represent data for boys' grades, and red points represent data for girls' grades.

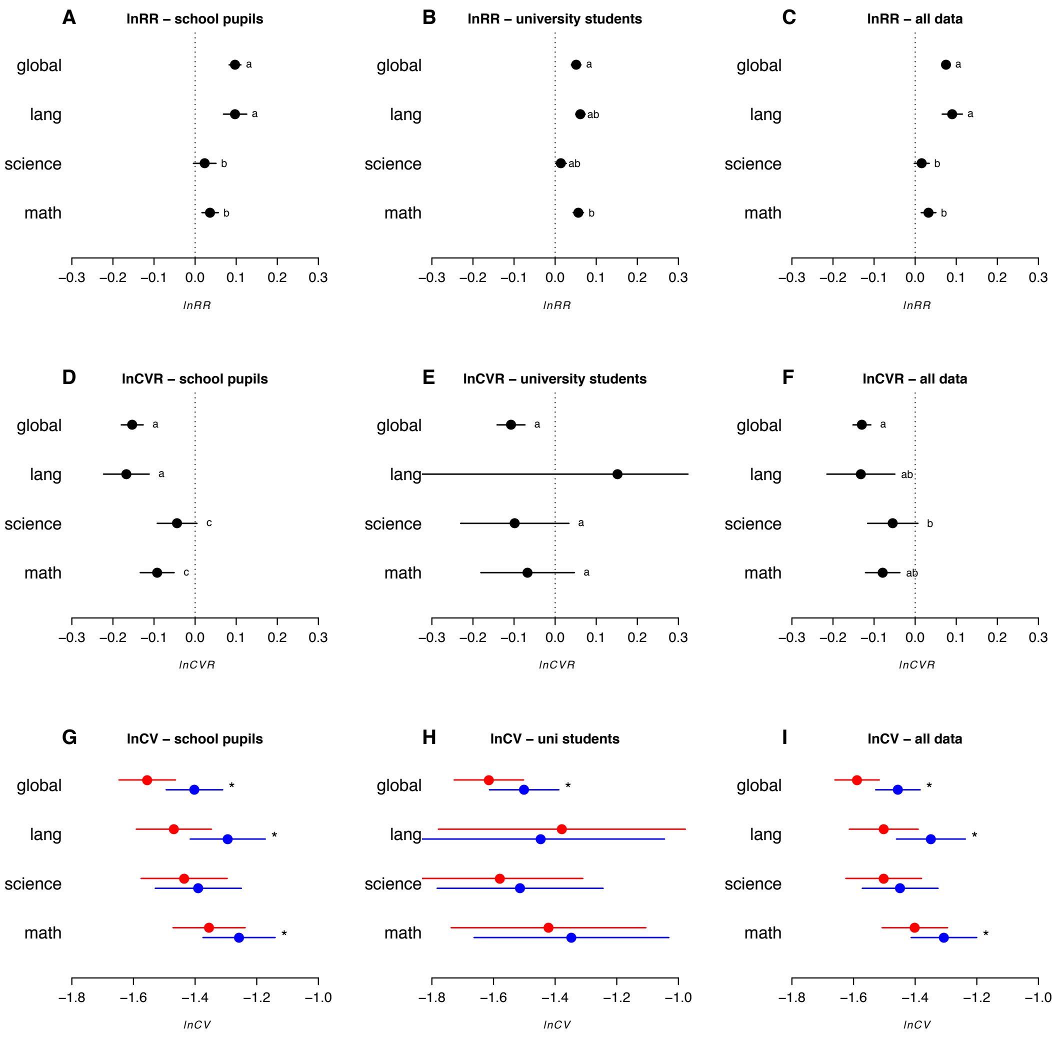

Supplementary Figure 10

Meta-analytic estimates of mean effect sizes (circles), and their 95% confidence intervals (whiskers), for gender differences in school grades according to subject type. First column: panels **A**, **D** and **G** – the school data subset. Second column: panels **B**, **E** and **H** – university data subset. Third column: panels **C**, **F** and **I** – whole sample. Blue represents data for boys' grades, and red represents data for girls' grades. Positive values of *lnRR* indicate lower male mean grades. Negative values of *lnCVR* indicate greater male variability in grades. Decreasing (more negative) values of *lnCV* indicate reduced variance.

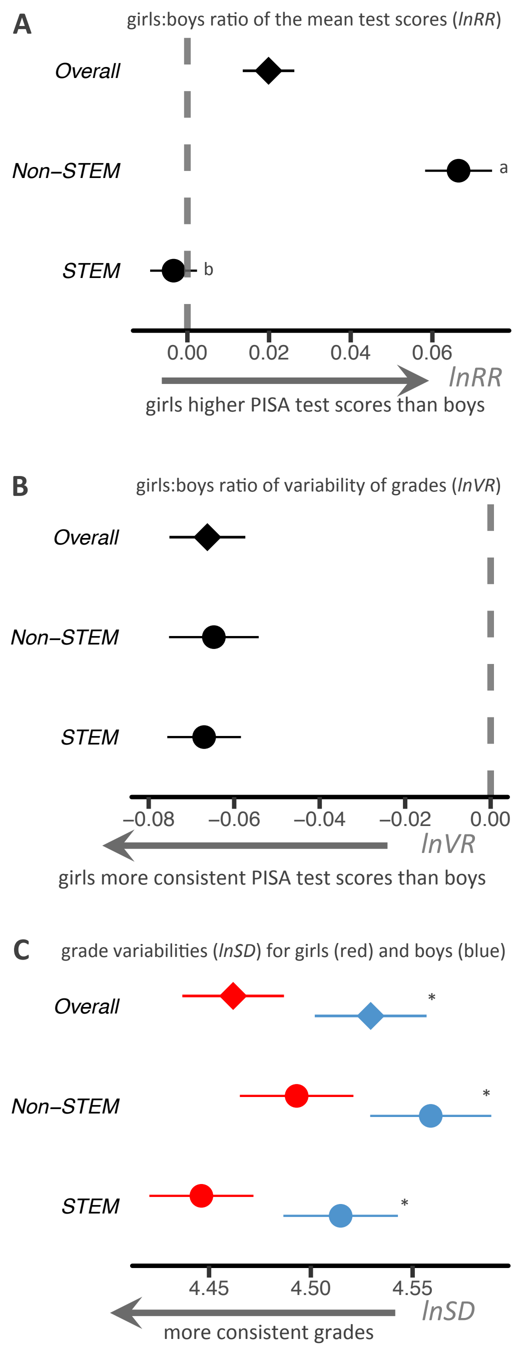

Supplementary Figure 11

Results of analyses on (**A**) ratios of the grade means, (**B**) ratios of grade variabilities, and (C) coefficients of variations for girls (red) and boys (blue) from the 2015 PISA, corresponding to Supplementary Table 14. Diamonds and circles represent meta-analytic estimates of mean effect sizes, and their 95% confidence intervals are drawn as whiskers. In panel **A**, natural logarithm of response ratio (*lnRR*) represents the

average difference between girls' and boys' test scores; positive values of *lnRR* indicate lower boys' tests scores. In panel **B**, natural logarithm of variation ratio (*lnVR*) represents the average difference in test score variation between boys and girls; negative values of *lnVR* indicate greater male variance. In panel **C**, natural logarithms of standard deviation in test scores (*lnSD*) are shown for girls and boys to illustrate grade variation by gender; more negative values of *lnSD* indicate less variation.

Supplementary Tables

Supplementary Table 1

Additional moderator variables in the dataset. A full list of extracted and coded variables can be downloaded from a dedicated repository on the Open Science Framework [16].

Variable	Extracted by Voyer & Voyer (2014)	Type	Categories	Description
Study_ID	No	Categorical	Unique ID for each study	Used to identify each study in the dataset
Cohort_ID	No	Categorical	Unique ID for each cohort	Used to identify each cohort to avoid pseudoreplication, e.g., 1 study reports scores for mathematics and language for the same group of students
Published	Yes	Categorical	Yes	Study is published
			No	Study is not published
Nationality	Yes	Categorical	North America	United States, Canada
			Other	Everywhere else
Year of publication	Yes	Continuous		Study publication year
Year of study	no	Continuous		Recorded when reported by the study, otherwise left blank. When multiple years are covered (such as a high school GPA), the graduation year was recorded for school samples, and the most recent enrolment year was recorded for university samples
Racial composition	yes	Categorical	White	>/= 75% White US sample
			Black	>/= 75% Black US sample
			Hispanic	>/= 75% Hispanic US sample
			Asian	>/= 75% Asian US sample
			Diverse	No racial majority US sample
			Not Reported	Race not reported for US

				sample
			non-U.S.	Sample not from US
Male to female ratio	yes	Categorical	m>f	More males than females in the cohort
			f=m	Equal numer of males and females in the cohort
			f>m	More females than males in the cohort
			Estimated	Sample sizes not split by gender, so equal sex ratio is assumed in the cohort
Sex ratio	no	Continuous		number of females/number of males in the cohort
Source of data	yes	Categorical	Elementary	Grades 1-6, ages 6-12
			Junior/middle	Grades 7-8, ages 13-14
			High school	Grades 9-12, ages 15-18
			Undergraduate	University / college
			Graduate	Post-graduate university / college
Mean age	no	Continuous		Where the mean age is reported, that value was extracted. For school samples, the mean age was estimated based on the grade level (e.g. year 4 students are recorded as 9.5 years). Age is not estimated for university samples unless explicitly reported by the study.

Supplementary Table 2

Estimated effect sizes for meta-analytic (intercept-only) models for grade difference between girls and boys, showing results for both variance (*lnCVR*) and mean differneces (*lnRR* and *SMD*). Negative *lnCVR* values can be interpreted as girls having less variable grades than boys. Positive values of *lnRR* and *SMD* can be interpreted as girls having higher grades than boys. Effects with confidence intervals (CI) not crossing zero are indicated in bold. Sigma^2 for cohort_ID is the amount of variance attributed to cohort identity (N levels = number of cohorts), and Sigma^2 for comp_ID represents residual variance for each unit of analysis (N levels = number of comparisons).

**Measure**	**Data**		**Fixed effects**			**Random effects**		
			Mean	CI.lb	CI.ub		Sigma^2	N levels
	School subset							
lnCVR		**Intercept**	**-0.131**	**-0.154**	**-0.109**	cohort_ID	0.00	141
						comp_ID	0.02	215
lnRR		**Intercept**	**0.074**	**0.061**	**0.086**	cohort_ID	0.00	141
						comp_ID	0.01	215
SMD		**Intercept**	**0.330**	**0.280**	**0.38**	cohort_ID	0.01	141
						comp_ID	0.08	215
	University subset							
lnCVR		**Intercept**	**-0.09**	**-0.122**	**-0.059**	cohort_ID	0.00	122
						comp_ID	0.03	131
lnRR		**Intercept**	**0.044**	**0.034**	**0.055**	cohort_ID	0.00	122
						comp_ID	0.00	131
SMD		**Intercept**	**0.213**	**0.162**	**0.264**	cohort_ID	0.07	122
						comp_ID	0.00	131
	All data							
lnCVR		**Intercept**	**-0.114**	**-0.133**	**-0.095**	cohort_ID	0.00	261

					comp_ID	0.02	346
lnRR	**Intercept**	**0.061**	**0.052**	**0.070**	cohort_ID	0.00	261
					comp_ID	0.01	346
SMD	**Intercept**	**0.279**	**0.243**	**0.315**	cohort_ID	0.01	261
					comp_ID	0.07	346

Supplementary Table 3

Estimated effect sizes for univariate meta-regression models with student population type (school pupils vs. university students) as a moderator. *lnCVR* is a measure of grade consistency of girls and boys and *lnRR* and *SMD* are measures of mean grade difference between girls and boys. Negative *lnCVR* values can be interpreted as girls having less variable grades than boys and positive *lnRR* and *SMD* values can be interpreted as girls having higher grades than boys. Effects with confidence intervals (CI) not crossing zero are indicated in bold. Effects with confidence intervals (CI) not crossing zero are indicated in bold. Number of effect sizes within each subset: school = 215; university = 131.

**Measure**	**Data**	**Fixed effects**		
		Mean	CI.lb	CI.ub
lnCVR				
	**School intercept**	**-0.134**	**-0.157**	**-0.112**
	**University intercept**	**-0.087**	**-0.117**	**-0.057**
	**School – University contrast**	**0.041**	**0.002**	**0.080**
lnRR				
	**School intercept**	**0.076**	**0.064**	**0.088**
	**University intercept**	**0.045**	**0.034**	**0.056**
	**School – University contrast**	**-0.028**	**-0.044**	**-0.011**
SMD				
	**School intercept**	**0.339**	**0.289**	**0.388**
	**University intercept**	**0.213**	**0.161**	**0.264**
	**School – University contrast**	**-0.118**	**-0.188**	**-0.047**

Supplementary Table 4

Estimated effect sizes for univariate meta-regression models with study year as a moderator. *lnCVR* is a measure of grade consistency of girls and boys and *lnRR* and *SMD* are measures of mean grade difference between girls and boys. Positive slope values can be interpreted as the difference between mean grades of boys and girls increasing over historical time. Estimates with confidence intervals (CI) not crossing zero are indicated in bold. “N missing” indicates how many missing values of study year moderator had to be imputed.

**Measure**	**Data**		**Fixed effects**			
			Mean	CI.lb	CI.ub	N missing
lnCVR	School subset					
		Study year	-0.001	-0.003	0.001	121
		Study year scaled	-0.029	-0.083	0.025	121
lnCVR	University subset					
		Study year	-0.002	-0.005	0.001	52
		Study year scaled	-0.017	-0.053	0.019	52
lnCVR	All data					
		Study year	-0.001	-0.003	0.001	173
		Study year scaled	-0.020	-0.052	0.013	173
lnRR	School subset					
		Study year	0.000	0.000	0.001	121
		Study year scaled	0.019	-0.017	0.055	121
lnRR	University subset					
		Study year	-0.001	-0.002	0.000	52
		Study year scaled	-0.010	-0.028	0.008	52
lnRR	All data					
		Study year	0.000	-0.001	0.001	173
		Study year scaled	0.005	-0.015	0.026	173
SMD	School subset					
		Study year	0.002	-0.001	0.006	121
		Study year scaled	0.067	-0.018	0.152	121
SMD	University subset					

		Study year	-0.001	-0.005	0.004	52
		Study year scaled	-0.007	-0.065	0.050	52
SMD	All data					
		Study year	0.001	-0.001	0.003	173
		Study year scaled	0.031	-0.010	0.073	173

Supplementary Table 5

Estimated slopes from univariate meta-regression models with sex and study year as moderators (with the interaction) for variation in grades of girls and boys (*lnCV*), using imputed data. Estimates with confidence intervals (CI) not crossing zero are indicated in bold. *k* – number of effect sizes included in the analysis.

**Measure**	**Data**		**Fixed effects**			
			Mean	CI.lb	CI.ub	*k*
lnCV	School subset					430
		Study year: girls	-0.002	-0.006	0.002	
		Study year scaled: girls	-0.064	-0.154	0.026	
		Study year: boys	-0.001	-0.005	0.003	
		Study year scaled: boys	-0.032	-0.122	0.058	
		Study year: boys-girls	0.001	0.000	0.002	
		**Study year scaled: boys-girls**	**0.032**	**0.004**	**0.060**	
lnCV	University subset					262
		Study year: girls	-0.002	-0.006	0.002	
		Study year scaled: girls	-0.064	-0.154	0.026	
		Study year: boys	-0.001	-0.005	0.003	
		Study year scaled: boys	-0.032	-0.122	0.058	
		Study year: boys-girls	0.001	0.000	0.002	
		**Study year scaled: boys-girls**	**0.032**	**0.004**	**0.060**	
lnCV	All data					692
		Study year: girls	-0.002	-0.006	0.002	
		Study year scaled: girls	-0.064	-0.154	0.026	
		Study year: boys	-0.001	-0.005	0.003	
		Study year scaled: boys	-0.032	-0.122	0.058	
		Study year: boys-girls	0.001	0.000	0.002	
		**Study year scaled: boys-girls**	**0.032**	**0.004**	**0.060**	

Supplementary Table 6

Estimated effect sizes for univariate meta-regression models with mean age of students as a moderator. *lnCVR* is a measure of grade consistency of girls and boys and *lnRR* and *SMD* are measures of mean grade difference between girls and boys. Positive slope values can be interpreted as the difference between mean grades of boys and girls increasing over historical time. Estimates with confidence intervals (CI) not crossing zero are indicated in bold. “N missing” indicates how many missing values of study year moderator had to be imputed.

**Measure**	**Data**		**Fixed effects**			
			Mean	CI.lb	CI.ub	N missing
lnCVR	School subset					
		Age	0.010	-0.001	0.021	37
		Age scaled	0.028	-0.003	0.060	37
lnCVR	University subset					
		Age	0.000	-0.020	0.019	91
		Age scaled	-0.002	-0.065	0.061	91
lnCVR	All data					
		Age	0.004	0.000	0.009	128
		Age scaled	0.018	-0.005	0.041	128
lnRR	School subset					
		Age	0.002	-0.003	0.007	37
		Age scaled	0.006	-0.008	0.019	37
lnRR	University subset					
		Age	-0.002	-0.010	0.006	91
		Age scaled	-0.006	-0.034	0.022	91
lnRR	All data					
		**Age**	**-0.002**	**-0.004**	**-0.001**	128
		**Age scaled**	**-0.011**	**-0.020**	**-0.003**	128
SMD	School subset					
		Age	0.015	-0.006	0.036	37
		Age scaled	0.043	-0.019	0.105	37
SMD	University subset					
		Age	0.003	-0.025	0.031	91
		Age scaled	0.014	-0.079	0.107	91
SMD	All data					
		Age	-0.006	-0.014	0.002	128
		Age scaled	-0.020	-0.061	0.021	128

414

Supplementary Table 7

Estimated slopes from univariate meta-regression models with sex and student mean age as moderators (with the interaction) for variation in grades of girls and boys (*lnCV*), using imputed data. Estimates with confidence intervals (CI) not crossing zero are indicated in bold. *k* – number of effect sizes included in the analysis.

**Measure**	**Data**		**Fixed effects**			
			Mean	CI.lb	CI.ub	*k*
lnCV	School subset					430
		Age: girls	0.004	-0.023	0.030	
		Age scaled: girls	0.010	-0.067	0.087	
		Age: boys	-0.009	-0.035	0.018	
		Age scaled: boys	-0.025	-0.101	0.052	
		**Age: boys-girls**	**-0.012**	**-0.022**	**-0.002**	
		**Age scaled: boys-girls**	**-0.035**	**-0.062**	**-0.007**	
lnCV	University subset					262
		**Age: girls**	**-0.054**	**-0.100**	**-0.008**	
		**Age scaled: girls**	**-0.188**	**-0.327**	**-0.049**	
		**Age: boys**	**-0.053**	**-0.092**	**-0.015**	
		**Age scaled: boys**	**-0.185**	**-0.305**	**-0.066**	
		Age: boys-girls	0.000	-0.018	0.019	
		Age scaled: boys-girls	0.003	-0.057	0.062	
lnCV	All data					692
		Age: girls	**-0.017**	**-0.031**	**-0.004**	
		Age scaled: girls	**-0.102**	**-0.169**	**-0.035**	
		**Age: boys**	**-0.022**	**-0.035**	**-0.008**	
		**Age scaled: boys**	**-0.122**	**-0.188**	**-0.056**	
		Age: boys-girls	-0.005	-0.009	0.000	
		Age scaled: boys-girls	-0.020	-0.041	0.001	

Supplementary Table 8

Estimated effect sizes for univariate meta-regression models with subjects (STEM, non-STEM, etc.) as a moderator for mean grade difference between girls and boys (*lnRR* and *SMD*). Positive values can be interpreted as girls having higher grades than boys. Effects with confidence intervals (CI) not crossing zero are indicated in bold. *k* – number of effect sizes included.

**Measure**	**Data**		**Fixed effects**			
			Mean	CI.lb	CI.ub	*k*
lnRR	School subset					215
		**Subject: STEM**	**0.031**	**0.011**	**0.051**	
		**Subject: Non-STEM**	**0.075**	**0.049**	**0.102**	
		**Subject: Global**	**0.098**	**0.084**	**0.113**	
		**Subject: Other/NR**	**0.115**	**0.012**	**0.218**	
		**non-STEM - STEM difference**	**-0.044**	**-0.065**	**-0.024**	
lnRR	University subset					131
		**Subject: STEM**	**0.024**	**0.002**	**0.045**	
		**Subject: Non-STEM**	**0.038**	**0.012**	**0.064**	
		**Subject: Global**	**0.054**	**0.042**	**0.066**	
		Subject: Other/NR	-0.001	-0.025	0.023	
lnRR	All data					346
		**Subject: STEM**	**0.025**	**0.009**	**0.041**	
		**Subject: Non-STEM**	**0.065**	**0.041**	**0.089**	
		**Subject: Global**	**0.077**	**0.067**	**0.087**	
		Subject: Other/NR	0.031	-0.015	0.077	
SMD	School subset					215
		**Subject: STEM**	**0.132**	**0.069**	**0.194**	
		**Subject: Non-STEM**	**0.356**	**0.269**	**0.443**	
		**Subject: Global**	**0.438**	**0.366**	**0.51**	

		**Subject: Other/NR**	**0.491**	**0.196**	**0.785**	
		**non-STEM - STEM difference**	**-0.224**	**-0.303**	**-0.146**	
SMD	University subset					131
		**Subject: STEM**	**0.104**	**0.007**	**0.201**	
		Subject: Non-STEM	0.119	-0.02	0.259	
		**Subject: Global**	**0.261**	**0.204**	**0.318**	
		Subject: Other/NR	0.031	-0.168	0.229	
SMD	All data					346
		**Subject: STEM**	**0.109**	**0.055**	**0.163**	
		**Subject: Non-STEM**	**0.307**	**0.225**	**0.389**	
		**Subject: Global**	**0.349**	**0.302**	**0.396**	
		Subject: Other/NR	0.163	-0.057	0.383	

Supplementary Table 9

Estimated effect sizes for univariate meta-regression models with subject (STEM, non-STEM, etc.) as a moderator for grade consistency of girls and boys (*lnCVR*). Negative *lnCVR* values can be interpreted as girls having less variable grades than boys. Effects with confidence intervals (CI) not crossing zero are indicated in bold. *k* – number of effect sizes included.

**Measure**	**Data**		**Fixed effects**			
			Mean	CI.lb	CI.ub	*k*
lnCVR	School subset					215
		**Subject: STEM**	**-0.079**	**-0.115**	**-0.043**	
		**Subject: Non-STEM**	**-0.149**	**-0.199**	**-0.099**	
		**Subject: Global**	**-0.153**	**-0.179**	**-0.126**	
		**Subject: Other/NR**	**-0.343**	**-0.546**	**-0.141**	
		**non-STEM - STEM difference**	**0.070**	**0.028**	**0.111**	
lnCVR	University subset					131
		Subject: STEM	-0.071	-0.144	0.001	
		Subject: Non-STEM	0.044	-0.259	0.347	
		**Subject: Global**	**-0.114**	**-0.148**	**-0.079**	
		Subject: Other/NR	-0.007	-0.115	0.101	
lnCVR	All data					346
		**Subject: STEM**	**-0.075**	**-0.109**	**-0.042**	
		**Subject: Non-STEM**	**-0.121**	**-0.186**	**-0.055**	
		**Subject: Global**	**-0.132**	**-0.154**	**-0.110**	
		Subject: Other/NR	-0.091	-0.218	0.036	

Supplementary Table 10

Estimated effect sizes for univariate meta-regression models with subject (STEM, non-STEM, etc.) and sex as moderators (with the interaction) for variation in grades of girls and boys (*lnCV*). Intercept values are presented for each sex-subject combination separately alongside with girls-boys contrast for each subject. Effects with confidence intervals (CI) not crossing zero are indicated in bold. *k* – number of effect sizes included in the analysis.

Measure	Data		Fixed effects			
			Mean	CI.lb	CI.ub	*k*
lnCV	School subset:					432
	girls intercepts					
		**Subject: STEM**	**-1.371**	**-1.471**	**-1.270**	
		**Subject: Non-STEM**	**-1.472**	**-1.591**	**-1.353**	
		**Subject: Global**	**-1.556**	**-1.651**	**-1.460**	
		**Subject: Other/NR**	**-1.547**	**-2.210**	**-0.883**	
	boys intercepts					
		**Subject: STEM**	**-1.292**	**-1.393**	**-1.190**	
		**Subject: Non-STEM**	**-1.322**	**-1.451**	**-1.193**	
		**Subject: Global**	**-1.402**	**-1.500**	**-1.305**	
		**Subject: Other/NR**	**-1.287**	**-1.939**	**-0.634**	
	girls-boys difference					
		**Subject: STEM**	**0.079**	**0.040**	**0.118**	
		**Subject: Non-STEM**	**0.150**	**0.093**	**0.207**	
		**Subject: Global**	**0.153**	**0.126**	**0.180**	
		**Subject: Other/NR**	**0.260**	**0.130**	**0.390**	
lnCV	University subset:					262
	girls intercepts					
		**Subject: STEM**	**-1.514**	**-1.713**	**-1.315**	
		**Subject: Non-STEM**	**-1.358**	**-1.755**	**-0.960**	

		**Subject: Global**	**-1.615**	**-1.723**	**-1.507**	
		**Subject: Other/NR**	**-2.071**	**-2.579**	**-1.563**	
	boys intercepts					
		**Subject: STEM**	**-1.443**	**-1.633**	**-1.253**	
		**Subject: Non-STEM**	**-1.403**	**-1.562**	**-1.245**	
		**Subject: Global**	**-1.501**	**-1.606**	**-1.397**	
		**Subject: Other/NR**	**-2.063**	**-2.629**	**-1.498**	
	girls-boys difference					
		Subject: STEM	0.070	-0.003	0.144	
		Subject: Non-STEM	-0.046	-0.356	0.265	
		**Subject: Global**	**0.114**	**0.079**	**0.149**	
		Subject: Other/NR	0.008	-0.104	0.119	
lnCV	All data					694
	girls intercepts					
		**Subject: STEM**	**-1.433**	**-1.525**	**-1.341**	
		**Subject: Non-STEM**	**-1.511**	**-1.628**	**-1.394**	
		**Subject: Global**	**-1.588**	**-1.658**	**-1.518**	
		**Subject: Other/NR**	**-1.689**	**-2.207**	**-1.172**	
	boys intercepts					
		**Subject: STEM**	**-1.357**	**-1.452**	**-1.261**	
		**Subject: Non-STEM**	**-1.388**	**-1.503**	**-1.274**	
		**Subject: Global**	**-1.456**	**-1.526**	**-1.386**	
		**Subject: Other/NR**	**-1.605**	**-2.185**	**-1.025**	
	girls-boys difference					
		**Subject: STEM**	**0.076**	**0.042**	**0.111**	
		**Subject: Non-STEM**	**0.123**	**0.054**	**0.191**	
		**Subject: Global**	**0.132**	**0.110**	**0.155**	
		Subject: Other/NR	0.085	-0.034	0.204	

Supplementary Table 11

Estimated effect sizes for meta-analytic (intercept-only) models for mean school grade difference between STEM and non-STEM subject grades (*lnRR*), for cohorts where we had grade data available for both STEM and non-STEM subjects. Negative mean *lnRR* estimates can be interpreted as STEM grades being on average lower than non-STEM. Effects with confidence intervals (CI) not crossing zero are indicated in bold. The data represents 30 cohorts of pupils from 25 studies.

**Measure**	**Model**		**Fixed effects**				**Random effects**	
			Mean	CI.lb	CI.ub		$Sigma^2$	N levels
lnRR	Meta-analysis							
		Intercept (girls + boys)	-0.040	-0.108	0.027	cohort_ID	0.033	30
						comp_ID	0.003	60
lnRR	Meta-regression							
		**Girls intercept**	**-0.069**	**-0.137**	**-0.001**	cohort_ID	0.034	30
		Boys intercept	-0.009	-0.077	0.060	comp_ID	0.001	60

Supplementary Table 12

Estimated effect sizes for univariate meta-regression models with publication year as a moderator. *lnCVR* is a measure of grade consistency of girls and boys and *lnRR* and *SMD* are measures of mean grade difference between girls and boys. Positive slope values can be interpreted as the difference between mean grades of boys and girls increasing over historical time. Estimates with confidence intervals (CI) not crossing zero are indicated in bold. “N missing” indicates how many missing values of study year moderator had to be imputed.

**Measure**	**Data**		**Fixed effects**			
			Mean	CI.lb	CI.ub	*k*
lnCVR	School subset					
		Publication year	-0.001	-0.002	0.001	215
lnCVR	University subset					
		Publication year	-0.002	-0.004	0.001	131
lnCVR	All data					
		Publication year	-0.001	-0.002	0.000	346
lnRR	School subset					
		Publication year	0.000	-0.001	0.002	215
lnRR	University subset					
		Publication year	0.000	-0.001	0.000	131
lnRR	All data					
		Publication year	0.000	-0.001	0.001	346
SMD	School subset					
		Publication year	0.002	-0.002	0.005	215
SMD	University subset					
		Publication year	0.000	-0.004	0.003	131
SMD	All data					
		Publication year	0.001	-0.001	0.004	346

Supplementary Table 13

Estimated effect sizes for univariate meta-regression models on gender differences in school grades among North Americans students, with race of students (>75% Black or White) as a moderator. *lnCVR* is a measure of grade consistency of girls and boys and *lnRR* and *SMD* are measures of mean grade difference between girls and boys. A positive contrast for *lnCVR* can be interpreted as White students having a smaller gender difference in grade variability than Black students, whereas a negative contrast for *lnRR* and *SMD* can be interpreted as White students having a smaller gender difference in mean grades than Black students. Estimates with confidence intervals (CI) not crossing zero are indicated in bold. *k* – number of effect sizes included.

**Measure**	**Data**		**Fixed effects**			
			Mean	CI.lb	CI.ub	*k*
lnCVR	North American School subset					51
		**Black**	**-0.174**	**-0.269**	**-0.079**	
		**White**	**-0.151**	**-0.203**	**-0.099**	
		Black-White Contrast	0.023	-0.085	0.132	
lnRR	North American School subset					51
		**Black**	**0.129**	**0.074**	**0.184**	
		**White**	**0.079**	**0.055**	**0.103**	
		Black-White Contrast	-0.049	-0.109	0.010	
SMD	North American School subset					51
		Black	**0.492**	**0.384**	**0.600**	
		White	**0.385**	**0.154**	**0.615**	
		Black-White Contrast	-0.108	-0.362	0.147	

Supplementary Table 14

Estimated effect sizes and heterogeneity estimates for meta-analytic (intercept-only) models, and meta-regression models with subject (STEM or Non-STEM) as moderators, for PISA test scores. *lnRR* is a measure of mean difference between test scores for girls and boys. *lnVR* is a measure of the difference in variability between girls and boys. *lnSD* is the total variability for each Jurisdiction. I^2_{Total} represents proportion of variance not attributed to standard error. $I^2_{Jurisdiction}$ represents proportion of variance attributed to the Jurisdiction where students were tested. $I^2_{comp\_ID}$ represents residuals against sampling error.

**Measure**	**Data**	**Fixed effects**			**Random effects**			**Heterogeneity**		
		Mean	CI.lb	CI.ub		Sigma^2	N levels	I^2_{Total}	$I^2_{Jurisdiction}$	$I^2_{comp\_ID}$
lnRR										
	**Intercept**	**0.020**	**0.014**	**0.026**	Jurisdiction	0.000	64	99.4	1.7	97.7
	**Subject: STEM**	**0.066**	**0.058**	**0.075**	comp_ID	0.002	192			
	Subject: Non-STEM	-0.003	-0.009	0.002						
	**non-STEM - STEM difference**	**-0.070**	**-0.074**	**-0.066**						
lnVR										
	**Intercept**	**-0.066**	**-0.075**	**-0.057**	Jurisdiction	0.001	64	79.3	75.3	4.0
	**Subject: STEM**	**-0.065**	**-0.075**	**-0.054**	comp_ID	0	192			
	**Subject: Non-STEM**	**-0.067**	**-0.076**	**-0.058**						
	non-STEM - STEM difference	-0.002	-0.008	0.003						
lnSD - overall										
	**non-STEM - STEM difference**	**4.526**	**4.499**	**4.554**	Jurisdiction	0.009	64	98.9	73.9	25.0
	**girls-boys difference**	**0.068**	**0.057**	**0.078**	comp_ID	0.003	192			

lnSD - girls				
	**non-STEM - STEM difference**	**-0.047**	**-0.062**	**-0.032**
lnSD - boys				
	**non-STEM - STEM difference**	**-0.044**	**-0.058**	**-0.030**

Supplementary Table 15

Estimated effect sizes for univariate meta-regression models with subject (classified as in Voyer & Voyer 2014) as a moderator for mean grade difference between girls and boys, using *lnRR* as the effect size. Positive *lnRR* values can be interpreted as girls having higher grades than boys. Effects with confidence intervals (CI) not crossing zero are indicated in bold. *k* – number of effect sizes included.

**Measure**	**Data**		**Fixed effects**			
			Mean	CI.lb	CI.ub	*k*
lnRR	School subset					215
		**Subject: Global**	**0.098**	**0.084**	**0.113**	
		**Subject: Language**	**0.091**	**0.059**	**0.123**	
		**Subject: Math**	**0.033**	**0.013**	**0.053**	
		Subject: Science	0.024	-0.006	0.055	
		**Subject: SocialSci**	**0.037**	**0.016**	**0.058**	
		**Subject: Other/NR**	**0.114**	**0.009**	**0.218**	
lnRR	University subset					131
		**Subject: Global**	**0.055**	**0.043**	**0.067**	
		**Subject: Language**	**0.050**	**0.026**	**0.075**	
		**Subject: Math**	**0.039**	**-0.004**	**0.082**	
		**Subject: Science**	**0.009**	**-0.008**	**0.025**	
		**Subject: SocialSci**	**0.012**	**-0.034**	**0.057**	
		Subject: Other/NR	-0.001	-0.025	0.022	
lnRR	All data					346
		**Subject: Global**	**0.076**	**0.066**	**0.086**	
		**Subject: Language**	**0.084**	**0.056**	**0.112**	
		**Subject: Math**	**0.029**	**0.011**	**0.048**	
		Subject: Science	0.017	-0.003	0.038	
		**Subject: SocialSci**	**0.024**	**0.004**	**0.044**	

Subject: Other/NR	0.031	-0.015	0.078

Supplementary Table 16

Estimated effect sizes for univariate meta-regression models with subject as a moderator for mean grade difference between girls and boys, using *SMD* as the effect size (for comparison with Voyer & Voyer 2017). Positive estimates can be interpreted as girls having higher grades than boys. Effects with confidence intervals (CI) not crossing zero are indicated in bold. *k* – number of effect sizes included.

**Measure**	**Data**		**Fixed effects**			
			Mean	CI.lb	CI.ub	*k*
SMD	School subset					215
		**Subject: Global**	**0.438**	**0.366**	**0.510**	
		**Subject: Language**	**0.415**	**0.314**	**0.515**	
		**Subject: Math**	**0.132**	**0.067**	**0.197**	
		**Subject: Science**	**0.120**	**0.012**	**0.228**	
		**Subject: SocialSci**	**0.201**	**0.126**	**0.276**	
		**Subject: Other/NR**	**0.496**	**0.192**	**0.801**	
SMD	University subset					131
		**Subject: Global**	**0.266**	**0.208**	**0.324**	
		Subject: Language	0.160	-0.041	0.362	
		Subject: Math	0.160	-0.032	0.351	
		Subject: Science	0.041	-0.053	0.134	
		Subject: SocialSci	0.015	-0.156	0.186	
		Subject: Other/NR	0.028	-0.169	0.225	
SMD	All data					346
		**Subject: Global**	**0.349**	**0.302**	**0.396**	
		**Subject: Language**	**0.381**	**0.287**	**0.474**	
		**Subject: Math**	**0.124**	**0.060**	**0.188**	
		**Subject: Science**	**0.080**	**0.002**	**0.159**	
		**Subject: SocialSci**	**0.140**	**0.062**	**0.219**	
		Subject: Other/NR	0.166	-0.058	0.390	

Supplementary Table 17

Estimated effect sizes for univariate meta-regression models with subject (classified as in Voyer & Voyer 2014) as a moderator for grade consistency of girls and boys (*lnCVR*). Negative *lnCVR* values can be interpreted as girls having less variable grades than boys. Effects with confidence intervals (CI) not crossing zero are indicated in bold. *k* – number of effect sizes included.

**Measure**	**Data**		**Fixed effects**			
			Mean	CI.lb	CI.ub	*k*
lnCVR	School subset					215
		**Subject: Global**	**-0.153**	**-0.180**	**-0.126**	
		**Subject: Language**	**-0.170**	**-0.226**	**-0.115**	
		**Subject: Math**	**-0.093**	**-0.134**	**-0.053**	
		Subject: Science	-0.042	-0.091	0.007	
		Subject: SocialSci	**-0.089**	**-0.150**	**-0.029**	
		**Subject: Other/NR**	**-0.339**	**-0.541**	**-0.137**	
lnCVR	University subset					131
		**Subject: Global**	**-0.114**	**-0.148**	**-0.079**	
		Subject: Language	0.068	-0.504	0.640	
		Subject: Math	-0.076	-0.205	0.053	
		Subject: Science	-0.068	-0.151	0.015	
		Subject: SocialSci	0.020	-0.209	0.250	
		Subject: Other/NR	-0.007	-0.116	0.102	
lnCVR	All data					346
		**Subject: Global**	**-0.132**	**-0.154**	**-0.110**	
		**Subject: Language**	**-0.149**	**-0.228**	**-0.071**	
		**Subject: Math**	**-0.090**	**-0.130**	**-0.049**	
		Subject: Science	-0.052	-0.099	-0.006	
		Subject: SocialSci	-0.058	-0.138	0.023	
		Subject: Other/NR	-0.089	-0.214	0.037	

501

Supplementary Table 18

Estimated effect sizes for univariate meta-regression models with subject (classified as in Voyer & Voyer 2014) and sex as moderators (with interaction) for variation in grades of girls and boys (*lnCV*). Intercept values are presented for each sex-subject combination separately alongside girls-boys contrast for each subject. Effects with confidence intervals (CI) not crossing zero are indicated in bold. *k* – number of effect sizes included in the analysis.

Measure	Data		Fixed effects			
			Mean	CI.lb	CI.ub	*k*
lnCV	School subset:					430
	girls intercepts					
		**Subject: Global**	**-1.555**	**-1.651**	**-1.458**	
		**Subject: Language**	**-1.466**	**-1.585**	**-1.346**	
		**Subject: Math**	**-1.352**	**-1.453**	**-1.250**	
		**Subject: Science**	**-1.433**	**-1.556**	**-1.310**	
		**Subject: SocialSci**	**-1.490**	**-1.660**	**-1.320**	
		**Subject: Other/NR**	**-1.561**	**-2.223**	**-0.899**	
	boys intercepts					
		**Subject: Global**	**-1.402**	**-1.500**	**-1.304**	
		**Subject: Language**	**-1.290**	**-1.428**	**-1.153**	
		**Subject: Math**	**-1.257**	**-1.361**	**-1.153**	
		**Subject: Science**	**-1.390**	**-1.512**	**-1.268**	
		**Subject: SocialSci**	**-1.406**	**-1.558**	**-1.254**	
		**Subject: Other/NR**	**-1.305**	**-1.961**	**-0.648**	
	girls-boys difference					
		**Subject: Global**	**0.153**	**0.125**	**0.181**	
		**Subject: Language**	**0.175**	**0.112**	**0.239**	

		**Subject: Math**	**0.095**	**0.052**	**0.137**	
		Subject: Science	0.044	-0.013	0.101	
		**Subject: SocialSci**	**0.084**	**0.011**	**0.157**	
		**Subject: Other/NR**	**0.257**	**0.127**	**0.386**	
lnCV	University subset:					262
	girls intercepts					
		**Subject: Global**	**-1.615**	**-1.725**	**-1.504**	
		**Subject: Language**	**-1.375**	**-2.012**	**-0.739**	
		**Subject: Math**	**-1.421**	**-1.757**	**-1.085**	
		**Subject: Science**	**-1.581**	**-1.849**	**-1.313**	
		**Subject: SocialSci**	**-1.382**	**-1.883**	**-0.881**	
		**Subject: Other/NR**	**-2.071**	**-2.587**	**-1.555**	
	boys intercepts					
		**Subject: Global**	**-1.501**	**-1.608**	**-1.394**	
		**Subject: Language**	**-1.447**	**-1.641**	**-1.253**	
		**Subject: Math**	**-1.347**	**-1.603**	**-1.090**	
		**Subject: Science**	**-1.514**	**-1.805**	**-1.222**	
		**Subject: SocialSci**	**-1.402**	**-1.692**	**-1.113**	
		**Subject: Other/NR**	**-2.063**	**-2.638**	**-1.489**	
	girls-boys difference					
		Subject: Global	**0.114**	**0.078**	**0.150**	
		Subject: Language	-0.071	-0.663	0.521	
		Subject: Math	0.074	-0.058	0.207	
		Subject: Science	0.067	-0.017	0.152	
		Subject: SocialSci	-0.020	-0.254	0.214	
		Subject: Other/NR	0.008	-0.106	0.121	
lnCV	All data					692
	girls intercepts					
		**Subject: Global**	**-1.588**	**-1.659**	**-1.517**	

	**Subject: Language**	**-1.498**	**-1.616**	**-1.380**
	**Subject: Math**	**-1.397**	**-1.495**	**-1.299**
	**Subject: Science**	**-1.500**	**-1.610**	**-1.391**
	**Subject: SocialSci**	**-1.522**	**-1.684**	**-1.359**
	**Subject: Other/NR**	**-1.691**	**-2.208**	**-1.175**
boys intercepts				
	**Subject: Global**	**-1.456**	**-1.526**	**-1.385**
	**Subject: Language**	**-1.346**	**-1.469**	**-1.222**
	**Subject: Math**	**-1.306**	**-1.403**	**-1.209**
	**Subject: Science**	**-1.449**	**-1.566**	**-1.331**
	**Subject: SocialSci**	**-1.466**	**-1.599**	**-1.332**
	**Subject: Other/NR**	**-1.607**	**-2.187**	**-1.027**
girls-boys difference				
	**Subject: Global**	**0.132**	**0.110**	**0.155**
	**Subject: Language**	**0.153**	**0.070**	**0.235**
	**Subject: Math**	**0.091**	**0.049**	**0.133**
	**Subject: Science**	**0.052**	**0.005**	**0.099**
	Subject: SocialSci	0.056	-0.029	0.140
	Subject: Other/NR	0.084	-0.036	0.203

509

Supplementary Table 19

Heterogeneity estimates for meta-analytic (intercept-only) models for overall grade consistency of girls and boys (*lnCVR*) and for overall mean grade difference between girls and boys (*lnRR* and *SMD*). Analyses were run on all included data and on two data subsets consisting of school pupils and university students, respectively. I^2_{Total} represents proportion of variance not attributed to standard error. $I^2_{\text{cohort\_ID}}$ represents proportion of variance attributed to cohort identity. $I^2_{\text{comp\_ID}}$ represents residuals against sampling error.

**Measure**	**Data**	**Heterogeneity**		
		I^2_{Total}	$I^2_{\text{cohort\_ID}}$	$I^2_{\text{comp\_ID}}$
	School subset			
lnCVR		98.4%	10.1%	88.3%
lnRR		99.0%	9.8%	89.2%
SMD		60.0%	9.0%	51.1%
	University subset			
lnCVR		99.5%	0.0%	99.5%
lnRR		99.2%	97.2%	2.1%
SMD		60.2%	57.5%	2.7%
	All data			
lnCVR		99.2%	4.0%	95.2%
lnRR		99.3%	11.0%	88.3%
SMD		61.3%	10.4%	51.0%

Supplementary Table 20

Estimated moderator effects from multivariate meta-regression models with study year, age and with subject (classified as in Voyer & Voyer 2014) as moderators for grade consistency of girls and boys (*lnCVR*). Negative *lnCVR* intercept values for different subjects can be interpreted as girls having less variable grades than boys. “Study year” and “Age” variables are scaled (*Z*-transformed). Effects with confidence intervals (CI) not crossing zero are indicated in bold. “N missing” indicates how many missing values of a moderator had to be imputed.

**Measure**	**Data**		**Fixed effects**			
			Mean	CI.lb	CI.ub	N missing
lnCVR	School subset					
		Study year (slope)	-0.035	-0.087	0.018	121
		Age (slope)	0.033	0.000	0.067	37
		**Subject: Global**	**-0.151**	**-0.184**	**-0.118**	
		**Subject: Language**	**-0.184**	**-0.247**	**-0.122**	
		**Subject: Maths**	**-0.113**	**-0.158**	**-0.067**	
		Subject: Other/NR	-0.368	-0.765	0.029	
		Subject: Science	-0.048	-0.099	0.003	
		Subject: SocialSci	-0.065	-0.197	0.067	
lnCVR	University subset					
		Study year (slope)	-0.025	-0.072	0.023	52
		Age (slope)	-0.013	-0.089	0.062	91
		**Subject: Global**	**-0.121**	**-0.155**	**-0.087**	
		Subject: Language	-0.086	-0.541	0.369	
		Subject: Maths	-0.074	-0.196	0.049	
		Subject: Other/NR	-0.007	-0.129	0.115	
		Subject: Science	-0.058	-0.142	0.026	
		Subject: SocialSci	0.012	-0.197	0.220	
lnCVR	All data					

Study year (slope)	-0.019	-0.050	0.011	173
Age (slope)	0.023	-0.004	0.051	128
**Subject: Global**	**-0.149**	**-0.173**	**-0.125**	
**Subject: Language**	**-0.159**	**-0.226**	**-0.093**	
**Subject: Maths**	**-0.107**	**-0.151**	**-0.063**	
Subject: Other/NR	-0.136	-0.276	0.004	
Subject: Science	-0.059	-0.110	-0.009	
Subject: SocialSci	-0.058	-0.165	0.049	

Supplementary Table 21

Estimated moderator effects from multivariate meta-regression models with study year, age and with subject (classified as in Voyer & Voyer 2014) as moderators for mean grade difference between girls and boys (*lnRR*). Positive *lnRR* intercept values for different subjects can be interpreted as girls having higher grades than boys. "Study year" and "Age" variables are scaled (*Z*-transformed). Effects with confidence intervals (CI) not crossing zero are indicated in bold. "N missing" indicates how many missing values of a moderator had to be imputed.

**Measure**	**Data**		**Fixed effects**			
			Mean	CI.lb	CI.ub	N missing
lnRR	School subset					
		Study year (slope)	0.019	-0.009	0.046	121
		Age (slope)	0.001	-0.013	0.016	37
		**Subject: Global**	**0.102**	**0.086**	**0.118**	
		**Subject: Language**	**0.102**	**0.065**	**0.139**	
		**Subject: Maths**	**0.050**	**0.024**	**0.075**	
		Subject: Other/NR	0.187	-0.220	0.594	
		**Subject: Science**	**0.032**	**0.008**	**0.055**	
		**Subject: SocialSci**	**0.055**	**0.025**	**0.084**	
lnRR	University subset					
		Study year (slope)	-0.011	-0.033	0.010	52
		Age (slope)	-0.011	-0.041	0.020	91
		**Subject: Global**	**0.066**	**0.051**	**0.081**	
		Subject: Language	0.026	-0.018	0.071	
		**Subject: Maths**	**0.026**	**-0.051**	**0.102**	
		Subject: Other/NR	0.007	-0.031	0.046	
		Subject: Science	0.014	-0.019	0.048	
		Subject: SocialSci	-0.026	-0.078	0.027	
lnRR	All data					
		Study year (slope)	0.004	-0.015	0.023	173

**Age (slope)**	**-0.017**	**-0.028**	**-0.005**	128
**Subject: Global**	**0.093**	**0.080**	**0.106**	
**Subject: Language**	**0.090**	**0.058**	**0.122**	
**Subject: Maths**	**0.043**	**0.018**	**0.068**	
Subject: Other/NR	0.086	-0.050	0.223	
**Subject: Science**	**0.029**	**0.007**	**0.052**	
**Subject: SocialSci**	**0.030**	**-0.001**	**0.061**	

Supplementary Table 22

Estimated moderator effects from multivariate meta-regression models with study year, age and with subject (classified as in Voyer & Voyer 2014) as moderators for mean grade difference between girls and boys (*SMD*). Positive *SMD* intercept values for different subjects can be interpreted as girls having higher grades than boys. “Study year” and “Age” variables are scaled (*Z*-transformed). Effects with confidence intervals (CI) not crossing zero are indicated in bold. “N missing” indicates how many missing values of a moderator had to be imputed. Results are not shown for the university subset, as this model did not converge.

**Measure**	**Data**		**Fixed effects**			
			Mean	CI.lb	CI.ub	N missing
SMD	School subset					
		Study year (slope)	0.051	-0.024	0.127	121
		Age (slope)	0.032	-0.034	0.098	37
		**Subject: Global**	**0.449**	**0.361**	**0.538**	
		**Subject: Language**	**0.425**	**0.321**	**0.529**	
		**Subject: Maths**	**0.160**	**0.088**	**0.232**	
		Subject: Other/NR	0.548	-0.324	1.421	
		**Subject: Science**	**0.119**	**0.005**	**0.233**	
		**Subject: SocialSci**	**0.211**	**0.084**	**0.337**	
SMD	University subset					
		Study year (slope)	0.011	-0.056	0.078	52
		Age (slope)	0.010	-0.089	0.109	91
		**Subject: Global**	**0.293**	**0.222**	**0.364**	
		Subject: Language	0.213	-0.006	0.433	
		Subject: Maths	0.180	-0.054	0.414	
		Subject: Other/NR	0.008	-0.228	0.245	
		Subject: Science	0.028	-0.140	0.196	

		Subject: SocialSci	0.061	-0.209	0.331	
SMD	All data					
		Study year (slope)	0.029	-0.012	0.070	173
		Age (slope)	-0.034	-0.079	0.010	128
		**Subject: Global**	**0.395**	**0.333**	**0.457**	
		**Subject: Language**	**0.394**	**0.296**	**0.492**	
		**Subject: Maths**	**0.140**	**0.064**	**0.215**	
		Subject: Other/NR	0.284	-0.044	0.612	
		Subject: Science	0.091	-0.001	0.183	
		**Subject: SocialSci**	**0.158**	**0.045**	**0.271**	

Supplementary Table 23

Estimated moderator effects from Bayesian multivariate meta-regression models with study year, age and subject (STEM, non-STEM, etc.) as moderators for grade consistency of girls and boys (*lnCVR*). Negative *lnCVR* intercept values for different subjects can be interpreted as girls having less variable grades than boys. "Study year" and "Age" variables are scaled (*Z*-transformed). Effects with Highest Posterior Density intervals (HPD) not crossing zero are indicated in bold.

**Measure**	**Data**		**Fixed effects**				
			Mode	Mean	SD	HPD.lb	HPD.ub
lnCVR	School subset						
		Study year (slope)	-0.033	-0.03	0.019	-0.064	0.007
		Age (slope)	-0.005	-0.012	0.025	-0.057	0.039
		Subject: STEM	-0.023	-0.007	0.068	-0.161	0.108
		Subject: Non-STEM	0.109	0.086	0.098	-0.089	0.289
		**Subject: Global**	**-0.121**	**-0.116**	**0.021**	**-0.156**	**-0.076**
		Subject: Other/NR	-0.059	-0.07	0.059	-0.182	0.041
lnCVR	University subset						
		Study year (slope)	-0.023	-0.03	0.018	-0.065	0.008
		Age (slope)	-0.016	-0.013	0.024	-0.061	0.034
		Subject: STEM	0.036	-0.005	0.069	-0.139	0.133
		Subject: Non-STEM	0.084	0.079	0.097	-0.102	0.278
		**Subject: Global**	**-0.113**	**-0.117**	**0.020**	**-0.154**	**-0.077**
		Subject: Other/NR	-0.046	-0.069	0.061	-0.184	0.051

**Measure**	**Data**	**Random effects**					
			Mode	Mean	SD	HPD.lb	HPD.ub
lnCVR	School subset						
		cohort_ID	0.000	0.002	0.002	0.000	0.007
		residuals	0.029	0.031	0.005	0.021	0.041

lnCVR	University subset						
		cohort_ID	0.000	0.002	0.002	0.000	0.007
		residuals	0.028	0.032	0.005	0.022	0.041

**Measure**	**Data**		**Heterogeneity**			**DIC**	
			Mode	Mean	SD	Mean	Range
lnCVR	School subset						
		I^2 cohort_ID	0.1	5.3	6.6	-55.08	(-55.19, -54.99)
		I^2 comp_ID	99.4	94.2	6.6		
		I^2 total	99.6	99.5	0.1		
lnCVR	University subset						
		I^2 cohort_ID	0.1	5.2	6.5	-55.08	(-55.19 , -54.99)
		I^2 comp_ID	99.4	94.3	6.5		
		I^2 total	99.6	99.5	0.1		

Supplementary Table 24

Estimated moderator effects from Bayesian multivariate meta-regression models with study year, age and subject (STEM, non-STEM, etc.) as moderators for mean grade difference between girls and boys (*lnRR*). Positive *lnRR* intercept values for different subjects can be interpreted as girls having higher grades than boys. “Study year” and “Age” variables are scaled (*Z*-transformed). Effects with Highest Posterior Density intervals (HPD) not crossing zero are indicated in bold.

**Measure**	**Data**		**Fixed effects**				
			Mode	Mean	SD	HPD.lb	HPD.ub
lnRR	School subset						
		Study year (slope)	-0.006	-0.009	0.006	-0.021	0.001
		Age (slope)	-0.012	-0.012	0.007	-0.025	0.001
		Subject: STEM	0.006	0.004	0.020	-0.035	0.042
		Subject: Non-STEM	0.037	0.042	0.029	-0.015	0.096
		**Subject: Global**	**0.057**	**0.056**	**0.006**	**0.044**	**0.068**
		**Subject: Other/NR**	**0.033**	**0.041**	**0.018**	**0.006**	**0.077**
lnRR	University subset						
		Study year (slope)	-0.006	-0.009	0.006	-0.021	0.001
		Age (slope)	-0.012	-0.012	0.007	-0.025	0.001
		Subject: STEM	0.006	0.004	0.020	-0.035	0.042
		Subject: Non-STEM	0.037	0.042	0.029	-0.015	0.096
		**Subject: Global**	**0.057**	**0.056**	**0.006**	**0.044**	**0.068**
		Subject: Other/NR	0.033	0.041	0.018	0.006	0.077

**Measure**	**Data**		**Random effects**				
			Mode	Mean	SD	HPD.lb	HPD.ub
lnRR	School subset						
		cohort_ID	0.000	0.001	0.001	0.000	0.003
		residuals	0.002	0.002	0.001	0.000	0.003

lnRR	University subset						
		cohort_ID	0.000	0.001	0.001	0.000	0.003
		residuals	0.002	0.002	0.001	0.000	0.003

**Measure**	**Data**		**Heterogeneity**			**DIC**	
			Mode	Mean	SD	Mean	Range
lnRR	School subset						
		I^2 cohort_ID	0.5	41.1	27.1	-443.75	(-445.66, -442.72)
		I^2 comp_ID	98.7	58.0	27.1		
		I^2 total	99.2	99.1	0.2		
lnRR	University subset						
		I^2 cohort_ID	0.5	41.1	27.1	-443.75	(-445.66, -442.72)
		I^2 comp_ID	98.7	58.0	27.1		
		I^2 total	99.2	99.1	0.2		

Supplementary Table 25

Intercept estimates from Bayesian Egger's regressions, performed on the full meta-regression model residuals and measurement errors from the school and university data subsets. Intercepts with Highest Posterior Density intervals (HPD) not crossing zero (in bold) indicate publication bias in the data.

**Test**	**Effect type**	**Data**	**Mean Intercept**	**HPD.lb**	**HPD.ub**
Egger's regression	lnCVR				
		School subset	-0.199	-0.664	0.275
		University subset	0.398	-1.173	2.169
Egger's regression	lnRR				
		School subset	-0.130	-0.693	0.438
		**University subset**	**0.860**	**0.126**	**1.520**
Egger's regression	SMD				
		School subset	0.029	-0.474	0.538
		University subset	0.294	-0.194	0.832

Supplementary References

1. Voyer, D. & Voyer, S. D. Gender differences in scholastic achievement: a meta-analysis. *Psychological Bulletin* **140,** 1174–1204 (2014).
2. Wallace, B. C., Small, K., Brodley, C. E., Lau, J. & Trikalinos, T. A. Deploying an interactive machine learning system in an Evidence-based Practice Center: Abstrackr. IHI '12 Proceedings of the 2nd ACM SIGHIT International Health Informatics Symposium, 819-824 (ACM Press, 2012). doi:10.1145/2110363.2110464
3. van Buuren, S. & Groothuis-Oudshoorn, K. mice: Multivariate imputation by chained equations in R. *Journal of Statistical Software* **45,** 1–67 (2011).
4. Fisher, Z. & Tipton, E. robumeta: An R-package for robust variance estimation in meta-analysis. 1–16 (2015).
5. Hadfield, J. D. MCMC methods for multi-response generalized linear mixed models: The MCMCglmm R package. *Journal of Statistical Software* **33,** 1–22 (2010).
6. Egger, M., Smith, G. D., Schneider, M. & Minder, C. Bias in meta-analysis detected by a simple, graphical test. *British Medical Journal* **315,** 629–634 (1997).
7. Nakagawa, S. & Santos, E. S. A. Methodological issues and advances in biological meta-analysis. *Evolutionary Ecology* **26,** 1253–1274 (2012).
8. French, M. T., Homer, J. F., Popovici, I. & Robins, P. K. What you do in high school matters: High School GPA, educational attainment, and labor market earnings as a young adult. *Eastern Economic Journal* **41,** 370–386 (2015).
9. Cohen, J. *F Tests on Means in the Analysis of Variance and Covariance, In Statistical Power Analysis for the Behavioral Sciences (Chapter 8 - Revised*

Edition). 273–406 (Academic Press, 1977).

10. Hedges, L. V. & Olkin, I. *Statistical Methods for Meta-Analysis*. (1985).

11. OECD. PISA 2015 Results (Volume I): Excellence and Equity in Education, PISA, OECD Publishing, Pairs, https://doi.org/10.1787/9789264266490-en, accessed on 14th June 2018. (2016).

12. OECD. PISA 2015 Database. OECD Skills Surveys, http://www.oecd.org/pisa/data/2015database/, accessed on 14th June 2018 (2015).

13. Nakagawa, S. *et al*. Meta-analysis of variation: Ecological and evolutionary applications and beyond. *Methods in Ecology and Evolution* **6,** 143–152 (2015).

14. Raudenbush, S. M. & Bryk, A. S. Examining correlates of diversity. *Journal of Educational Statistics* **12,** 241–269 (1987).

15. Viechtbauer, W. Conducting meta-analyses in R with the metafor package. *Journal of Statistical Software* **36,** 1–48 (2010).

16. O'Dea, R. E., Lagisz, M., Jennions, M. D. & Nakagawa, S. Data for "Gender differences in individual variation in academic grades fail to fit expected patterns for STEM." *Open Science Framework* osf.io/efm9t (2018).
