# Supplementary material for: Gender differences in individual variation in academic grades fail to fit expected patterns for STEM
Source: Nat Commun. 2018 Sep 25;9:3777. doi: 10.1038/s41467-018-06292-0 (PMC6156605; doi:10.1038/s41467-018-06292-0)
Supplement: Supplementary file 3 — Description of Additional Supplementary Files [file 41467_2018_6292_MOESM3_ESM.pdf]

Description of Additional Supplementary Files

File Name: Supplementary Data 1
Description: List of original studies reporting grades of boys and girls included in this metaanalysis. V&V 2014 – studies included in an earlier meta-analysis by Voyer 1, Geographic location – geographic region the data came from (North America versus Other locations), Academic level – whether data is for a school pupils (school) or university students (uni), k – number of effect sizes extracted from a given study.

File Name: Supplementary Data 2
Description: List of excluded full-text original publications that were deemed as potentially relevant after screening of titles, abstracts and keywords. Main reason for exclusion at full-text screening and extraction is provided.
